# Assessing agreement among alternative climate change projections to inform conservation recommendations in the contiguous United States

**DOI:** 10.1038/s41598-018-27721-6

**Published:** 2018-06-21

**Authors:** R. Travis Belote, Carlos Carroll, Sebastián Martinuzzi, Julia Michalak, John W. Williams, Matthew A. Williamson, Gregory H. Aplet

**Affiliations:** 1 The Wilderness Society, Bozeman, MT 59715 USA; 2 Klamath Center for Conservation Research, Orleans, CA 95556 USA; 3 Department of Forest and Wildlife Ecology, University of Wisconsin, Madison, WI 53706 USA; 4 School of Environmental and Forest Science, University of Washington, Seattle, WA 98195 USA; 5 Department of Geography and Center for Climatic Research, University of Wisconsin, Madison, WI 53706 USA; 6 Department of Environmental Science and Policy, University of California, Davis, CA 95616 USA; 7 The Wilderness Society, Denver, CO 80202 USA

## Abstract

Addressing uncertainties in climate vulnerability remains a challenge for conservation planning. We evaluate how confidence in conservation recommendations may change with agreement among alternative climate projections and metrics of climate exposure. We assessed agreement among three multivariate estimates of climate exposure (forward velocity, backward velocity, and climate dissimilarity) using 18 alternative climate projections for the contiguous United States. For each metric, we classified maps into quartiles for each alternative climate projections, and calculated the frequency of quartiles assigned for each gridded location (high quartile frequency = more agreement among climate projections). We evaluated recommendations using a recent climate adaptation heuristic framework that recommends emphasizing various conservation strategies to land based on current conservation value and expected climate exposure. We found that areas where conservation strategies would be confidently assigned based on high agreement among climate projections varied substantially across regions. In general, there was more agreement in forward and backward velocity estimates among alternative projections than agreement in estimates of local dissimilarity. Consensus of climate predictions resulted in the same conservation recommendation assignments in a few areas, but patterns varied by climate exposure metric. This work demonstrates an approach for explicitly evaluating alternative predictions in geographic patterns of climate change.

## Introduction

Ongoing changes in climate affect ecosystem composition, structure, and function^1–3^, and paleorecords clearly indicate a high sensitivity of species and ecosystem distributions to climate change^4,5^. Considerations of the ecological effects of future climate change create challenges for traditional conservation planning^6–10^. Conservation strategies focused on either restoration or preservation are being adjusted in light of climate impacts^10^. For instance, restoration ecologists increasingly consider future climatic conditions in planning^11^, and ecological reserves are proposed that consider projected impacts of climate change^12^. In addition, climate change and other human-caused stressors have resulted in calls to adjust management strategies in protected areas^13^.

**Figure 1:**
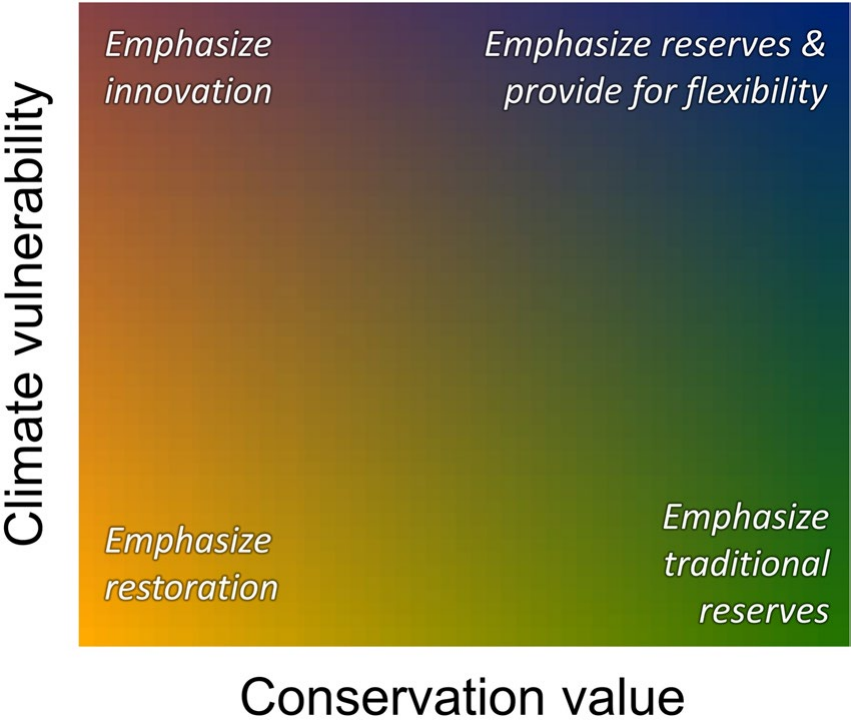
Conceptual framework proposed by Belote *et al*.^17^ that recommends conservation strategies be based on existing conservation value and projected climate vulnerability.

Faced with these challenges, climate adaptation heuristics to support conservation decisions have been developed using assessments of existing ecological conditions while considering the predicted or observed impacts of climate change^14–17^ (Fig. 1). These heuristic frameworks can guide management decisions, while also providing conceptual foundations to support mapped data indicating lands where various conservation strategies would reasonably be emphasized^16,17^. For instance, evaluation of predicted climate change impacts may result in land managers adjusting restoration strategies for areas with degraded ecological conditions. The historical range of variability may serve as an insufficient target for restoration when considering potential climate change impacts^18^. Similarly, decisions on protected areas designation locations or management can be revised based on ongoing or predicted changes in climate^13^, especially when climate-sensitive ecosystems or species occur within boundaries of such conservation reserves. Whether protected areas may require intensive management intervention, novel management options, or additional flexibility in a climate-altered future remains controversial, especially in relatively intact wildland ecosystems^19,20^. Climate adaptation heuristics have helped guide thinking on these controversies^10,14,17,21^ (Fig. 1).

Heuristic frameworks and maps typically rely on predictions of multivariate climate change without explicit regard for specific impacts to particular ecosystems or species^16,17^. Instead, heuristics rely on assumptions that evaluations of changes in multiple dimensions of climate may provide useful guidance on the relative vulnerability of species and ecosystems^22^. Climate vulnerability has been described as a function of climate exposure, sensitivity, and adaptive capacity^23^, though we focus here on climate exposure (i.e. the degree of climate change likely to be experienced by a species or locale). Climate exposure metrics include predictions of changes in individual climate variables^24^, geographic displacement of climate analogues (i.e., climate velocity)^25–27^, and multivariate shifts in climate^28,29^. Various metrics of predicted exposure suggest that different regions may experience relatively high degrees of climate change depending on which metric is evaluated^22^. These differences may reflect various ‘dimensions’ of climate change. Evaluating regions where high or low degrees of different exposure metrics coincide may be an important step in evaluating potential future impacts to complex ecosystems.

Heuristics for conservation using mapped indices of climate vulnerability also often focus on the central tendency of climate predictions by using data from multi-model ensembles^16^, median values of simulations^17^, or observed trends in recent historical climate^14^. Development of heuristics have been useful, but evaluating uncertainty in climate adaptation conservation planning remains a challenge^30–32^. Understanding the uncertainty in climate adaptation planning is critical because it affects confidence in conservation recommendations based on expected changes in climate. Variability in climate change projections can arise through various means including use of different baseline climatological data^33^, emissions scenarios or representative concentration pathways^28,34,35^, downscaling methods^36^, general circulation models (GCMs^37^), choice of climate vulnerability metrics^22,26,38^, and predicted ecological responses^30,32,39–43^, among others.

Our goal is to evaluate how confidence in conservation recommendations may vary when considering uncertainty in climate projections and variability in metrics of climate exposure, using the contiguous United States as a case study. We used a heuristic framework that recommends conservation strategies based on combinations of existing conservation value and projected climate vulnerability (Fig. 1^17^). To explicitly assess sources of uncertainty and their potential effects on confidence in conservation recommendations, we evaluated variability and agreement in predictions among general circulation models and greenhouse gas concentration scenarios, as well as different climate exposure metrics. We assumed that confidence in conservation recommendations increases with the agreement among climate scenarios and climate exposure metrics. Our specific objectives were to (1) map three multivariate metrics of climate exposure (forward climate velocity, backward climate velocity, and local climate dissimilarity); (2) map agreement among alternative climate projections for each of the three metrics of exposure; and (3) assess how agreement in climate projections influence confidence in assigning climate adaptation conservation strategies.

## Methods

### General approach

We relied on data available from the AdaptWest project, which is a collaborative effort to disseminate spatial data on predicted changes in climate to aid conservation planners (see adaptwest.databasin.org). We used 1-km resolution climate data from AdaptWest^38,44^ to derive three metrics of climate change exposure at one time step (30-year average centered on 2055). Specifically, we mapped estimates of (1) forward climate velocity, (2) backward climate velocity, and (3) local climate dissimilarity. These metrics have been used in previous studies to evaluate general measures of exposure not focused on any specific species or ecosystem, as is the case of our study^16,26,28^. For our analysis, we focused on multivariate metrics derived from the same data reduction method (principal components analysis) to emphasize variability derived from alternative climate projections, and not from other methodological decisions (i.e., which variables and approach to use to reduce data dimensionality). We also limited our analysis to one time step (predictions centered on the year 2055) to (1) reduce the complexity of our analysis, (2) focus on alternative predictions holding time constant, and (3) present results for a future timeframe likely to influence land management decisions.

We used nine climate simulations including projections from eight different general circulation models (GCMs) and one multi-model ensemble based on 15 different GCMs^44^; see Supplemental Table 1 for details of GCMs used. The multi-model ensemble includes some of the eight GCMs and so is not an independent case. However, it is included to represent the common usage of ensemble projections in conservation planning. These nine alternative climate projections allowed us to assess intermodel differences in climate process and parameterization schemes^37^. Additionally, we used predictions from two representative concentration pathways (RCP) of greenhouse gasses (i.e., RCP 4.5 or medium-low emissions, and RCP 8.5 or high emissions) that represent uncertainty due to policy choices over the coming decades. Predictions from RCP 4.5 are based on a trajectory of greenhouse gas concentrations that stabilizes with a net positive (warming) energetic forcing to the atmosphere of 4.5 W m^−2 ^^45^, while predictions from RCP 8.5 are based on a higher trajectory of greenhouse gas emissions that produces a greenhouse gas radiative forcing that is nearly twice that of RCP 4.5^46^. We pooled simulations representing the two RCP scenarios with the nine simulations from different GCMs, resulting in 18 different predictions for each of the three climate exposure metrics (Supplemental Figs 1–3). The purpose of producing these 18 different projections was to generate a range of reasonable climate predictions that represent alternatives available from the AdaptWest project for developing climate-informed conservation planning.

### Metric calculations

We calculated the three multivariate climate metrics of exposure for each of the 18 (2 RCPs × 9 GCMs) alternative climate projections using 11 climate variables^26^. The climate variables used were mean annual temperature (°C), mean temperature of the warmest month (MWMT, °C), mean temperature of the coldest month (MCMT, °C), difference between MCMT and MWMT (°C), mean annual precipitation (mm), mean summer (May to Sep) precipitation (mm), mean winter (Oct to Apr) precipitation (mm), degree-days above 5 °C (growing degree days), the number of frost-free days, Hargreave’s reference evaporation, and Hargreave’s climatic moisture index^47^. We followed Wang *et al*.^44^ in selecting bioclimatic variables based on previous studies concluding that these variables were important in ecological models (e.g., models separating forest ecosystems in British Columbia and the western United States^48^). These variables were transformed to meet assumptions of normality (log transform of precipitation and moisture index and square-root transform of degree days) and then subjected to a principal components analysis using a correlation matrix approach to reduce the dimensionality of the data. The first two principal component (PC) scores accounted for 89% of the variance in the multivariate climate space, with the PC1 and PC2 axes accounting for 66% and 23% of variance, respectively. PC1 was most strongly associated with temperature variables, and PC2 was most strongly associated with precipitation and moisture variables.

The first two PC scores were used to calculate three multivariate metrics of climate exposure: forward velocity, backward velocity, and local climate dissimilarity. Forward and backward climate velocities are correlated but distinct^26,38^. Forward and backward velocity measure the geographic displacement of climate analogues based on the first two PC scores, as a way of assessing the minimum distance organisms would need to travel to track changes in climate^26^ (but see^49^). Forward velocity is based on the distance that current climate conditions (average from 1981 to 2010) are projected to move from their current location into the future, whereas backward velocity measures the distance that climate conditions of the future are projected to have moved to arrive at their locations. Specifically, velocity estimates were calculated by first binning PC scores to map multivariate climate analogs for current and future time steps. Velocities were then calculated by measuring the minimum distance between climate analogs from current to future time steps (forward velocity) and future to current time steps (backward velocity).

Local climate dissimilarity, on the other hand, is a multivariate index that summarizes the magnitude of multivariate climate change expected at each grid cell. We estimated projected local climate dissimilarity for all grid cell locations by calculating Euclidean distances between current (based on average climate between 1981–2010) and future (2041–2070, i.e., centered on 2055) climate for the same grid cell in the first 2 principal components using:

$${\rm{Local}}\,{\rm{climate}}\,{\rm{dissimilarity}}=\sqrt{{({\rm{PC}}{1}_{future}-{\rm{PC}}{1}_{current})}^{2}+{({\rm{PC}}{2}_{future}-{\rm{PC}}{2}_{current})}^{2}}$$

This method is analogous to the one used by Williams *et al*.^28^, but we use the first two PC scores instead of climate variables, which is similar to Mahalanobis distances^29^.

### Agreement among climate exposure metrics and projections

We mapped agreement in climate predictions among the 18 different alternatives for each multivariate metric of climate exposure separately (Supplemental Figs 1–3). To do this, we classified the climate metric values for all 1-km grid cells into four quartiles and assigned each quartile an integer value of 1 (lower quartile) to 4 (upper quartile). This quartile assignment was done separately for each of the 18 simulations (Supplemental Fig. 4). Then, for each grid cell, we identified which quartile value was most frequently assigned to each grid cell (i.e., the mode) and the number of times the mode was recorded for each location (i.e., frequency of the mode) (Supplemental Fig. 4). The frequency of mode hence serves as an index of model agreement and uncertainty, with values ranging from 5 (minimum majority and little agreement among simulations) to 18 (perfect consensus). Other means to assess uncertainty include calculating the median and the standard deviation of estimates for all grid cell locations. We also calculated these values (Supplemental Fig. 5), but here focus on the mode, because maps derived from the heuristic framework of Belote *et al*.^17^ require classifying locations by their relative climate vulnerability. By calculating the mode of classified quartiles and the associated frequencies, we could more easily evaluate agreement in simulation predictions in the context of the value and vulnerability maps by which conservation strategies are recommended, compared to assessing variance among simulations.

To assess agreement among the three climate metrics, we overlaid the quartile modes (across all 18 simulations) for each metric and mapped the overlap between the three exposure metrics in the upper and lower quartile (see Supplemental Fig. 4 for more details). From this map we identified areas of metric agreement in assigning land to either low or high degree of vulnerability.

### Effect on conservation recommendations

Our approach is intended to explore differences in maps of conservation recommendations derived from the heuristic of Belote *et al*. (Fig. 1)^17^ using alternative climate change predictions. From this analysis, we ask how confident a conservation recommendation assignment would be based on alternative climate predictions. Confidence in recommendations would be greatest where multiple climate projections agree.

To evaluate how the level of agreement among climate projections and metrics influence confidence in assigning conservation strategies, we created bivariate maps using the quartile mode of each climate metric with a map of wildland conservation value as in Belote *et al*.^12^. Wildland conservation value is a composite map based on an assessment of human modification^50^, connectivity between protected areas^51^, and priorities for representing ecosystem^52^ and species diversity in conservation reserves^53^. By combining maps of this conservation value with future climate vulnerability, Belote *et al*.^17^ classify lands as either (1) high conservation value-high climate vulnerability, (2) high conservation value-low climate vulnerability, (3) low conservation value-low climate vulnerability, or (4) low conservation value-high climate vulnerability (Fig. 1). Such classification makes it possible to identify different conservation strategies, ranging from an emphasis on traditional reserve protection in high value-low vulnerability lands, to restoration to historical conditions in low value-low vulnerability locations, to innovative approaches that anticipate and manage for the future in low value-high vulnerability areas^17^. High value-high vulnerability areas represent challenging scenarios, whereby protection of conservation values is a priority, but expanded management flexibility may be required to allow interventions under a high degree of climate exposure (see^17^ for more discussion).

For the map of conservation values, we classified the composite wildland conservation value of Belote *et al*.^12^ into four quartiles and combined these quartiles with quartiles of climate vulnerability estimates described above. This resulted in a 4 × 4 classification of conservation value and climate vulnerability (based on exposure). We assess regions for assignment of conservation strategies with the highest levels of agreement among alternative climate projections by visually inspecting maps (*sensu*^54^) and quantifying the total area within each lower or upper quartile of climate metric and wildland conservation value (i.e., the corners of the bivariate legend).

We focused our attention on locations classified into the highest and lowest quartiles for both conservation value and climate vulnerability (i.e., corners of Fig. 1). The resulting bivariate maps show locations with high conservation value-low climate vulnerability; low conservation value-low climate vulnerability; low conservation value-high climate vulnerability; or high conservation value-high climate vulnerability. These are the lands where our confidence in assigning a conservation strategy based on values and vulnerability – per the heuristic framework – may be the highest when agreement in climate predictions is high. Specifically, we focus on these areas to remove sources of uncertainty associated with intermediate degrees of values and vulnerability. To assess confidence in the assignment of conservation recommendations, we mapped the level of agreement (i.e., number of times a cell was placed in the focal corner representing a conservation recommendation) and then calculated the land area for each level of agreement. This allowed us to evaluate how the total area of the classified value and vulnerability combinations (i.e., the “corners” of the heuristic framework and maps) change as the standard for “agreement” is raised from minimum majority (frequency ≥ 5 simulations) to full consensus (by mapping only grid cells assigned to the quartile mode for all 18 simulations, i.e., frequency = 18 simulations).

## Results

**Figure 2:**
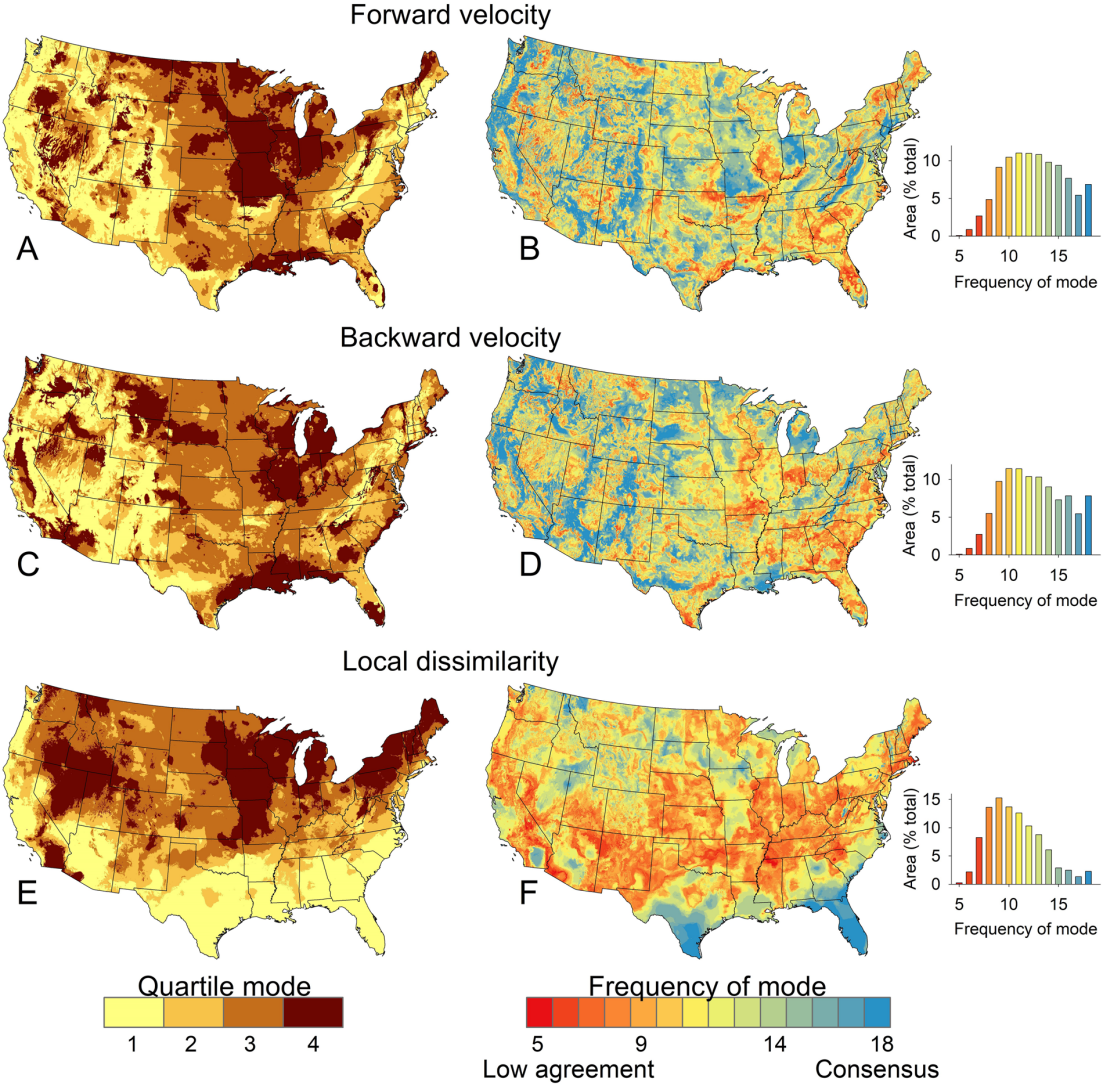
Mode of quartile in classified data of three climate vulnerability metrics and frequency of mode among 18 different projections for forward velocity (**A,B**), backward velocity (**C,D**), and climate dissimilarity (**E,F**). Maps B,D,F present an index of inter-simulation uncertainty for each climate metric, with areas of red indicating lower intermodel agreement and higher uncertainty.

Patterns of climate vulnerability based on the three metrics (forward velocity, backward velocity, and climate dissimilarity) varied throughout the country, as did the level of agreement among climate simulations for each metric (Fig. 2). For all three metrics, agreement in quartile mode classification was highest in the lower and upper quartiles; less agreement was observed in the middle quartiles. Velocity metrics tended to have higher agreement among projections than did estimates of climate dissimilarity based on frequency of quartile mode (see maps and histograms in Fig. 2). For instance, 82% and 80% of the total area was classified as the same quartile mode for forward and backward velocity in ≥10 of 18 projections, respectively, versus 60% of the total area that was classified as the same quartile mode in ≥10 simulations of the dissimilarity estimate (see histograms in Fig. 2). Similarly, 20% and 21% of the total area was classified as the same mode in ≥16 of 18 projections, whereas only 6% was classified as the same mode of dissimilarity. Full consensus among all 18 simulations occurred in 7%, 8%, and 2% of the U.S. based on quartile modes for estimates of forward velocity, backward velocity, and dissimilarity, respectively.

**Figure 3:**
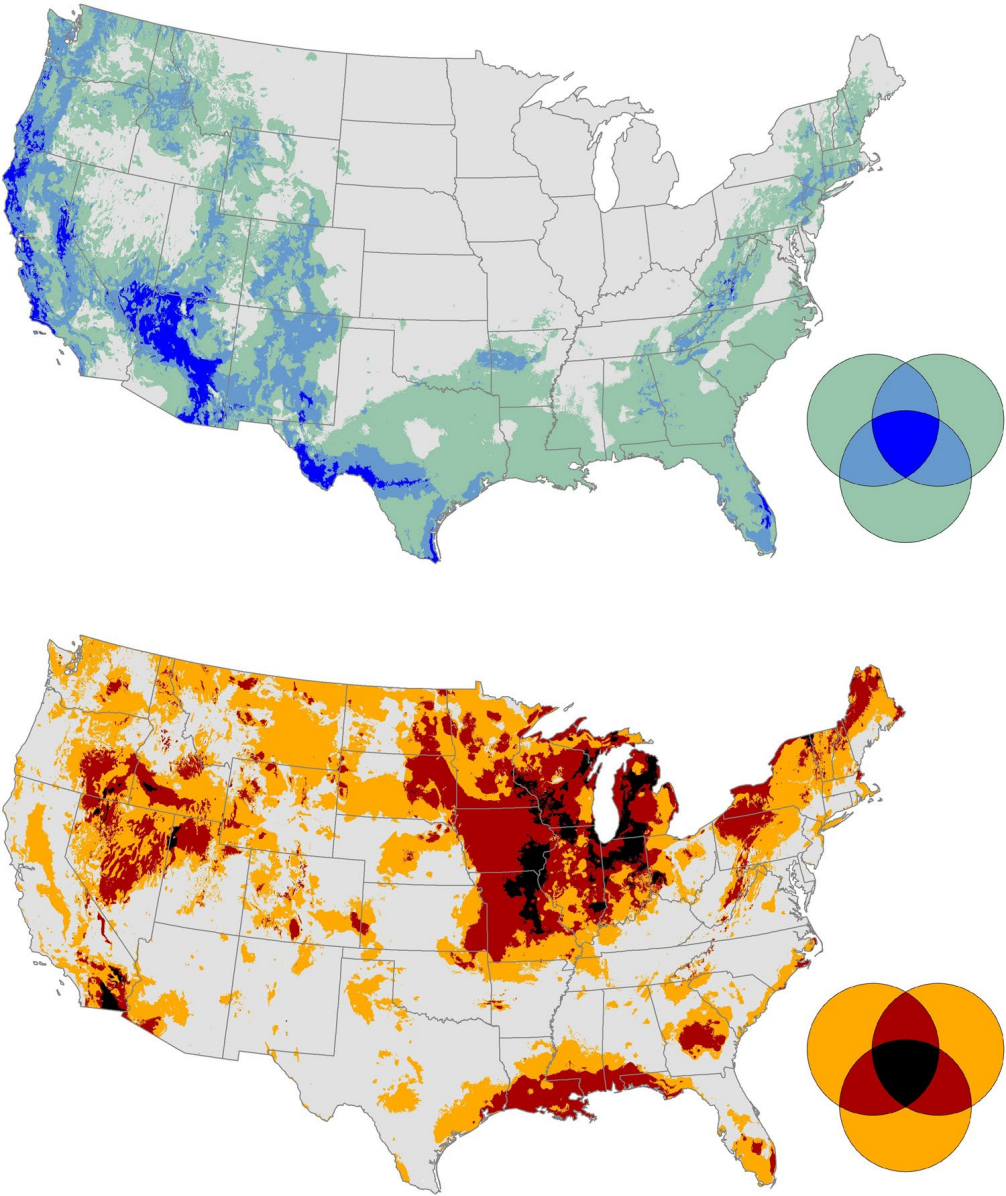
Maps of inter-metric agreement (or disagreement), showing where the mode of three climate metrics (forward velocity, backward velocity, and climate dissimilarity) were in the lower (top map) or upper (bottom map) quartile. The Venn diagram legends show areas where zero, one, two, or all three metrics were assigned an area to the lower or upper quartiles. In the top map, gray indicates areas where no metrics were in the lower quartile, green indicates where only one of the metrics were assigned to the lower quartile mode, light blue areas indicates two metrics had a mode in lower quartile mode, and dark blue indicates that all three metrics were assigned to the lower quartile mode. In the bottom map, the same pattern is used to map metric agreement using orange (one metric assigned to upper quartile mode), red (two metrics), and black (all three metrics assigned to upper quartile mode).

The upper Midwestern states were characterized by relatively high degree of climate exposure, whereas mountainous regions of the West and Appalachians were characterized by low forward and backward velocity with relatively high agreement among simulations. All three climate metrics result in low predicted exposure occurring in the coastal range of California, northern and central Arizona, and west Texas (Fig. 3). The upper quartile of all three climate metrics occurred in the upper Midwest and deserts and arid regions in the intermountain West. Areas where two of the three metrics agreed in relatively low or high predictions of exposure were common. Western mountains, the Appalachians, and much the coastal plain of the southeast were characterized by low exposure for at least 2 metrics. The Basin and Range of the West, the upper Midwest, and parts of the Gulf Coast were characterized by high exposure estimates in at least 2 metrics. Grid cells with full consensus among metrics classified into the lower quartile mode made up only 2.6% of the contiguous U.S. (dark blue areas in the upper map of Fig. 3), and grid cells with consensus in the upper quartile simulation made up 2.5% area (black areas in the lower map of Fig. 3).

**Figure 4:**
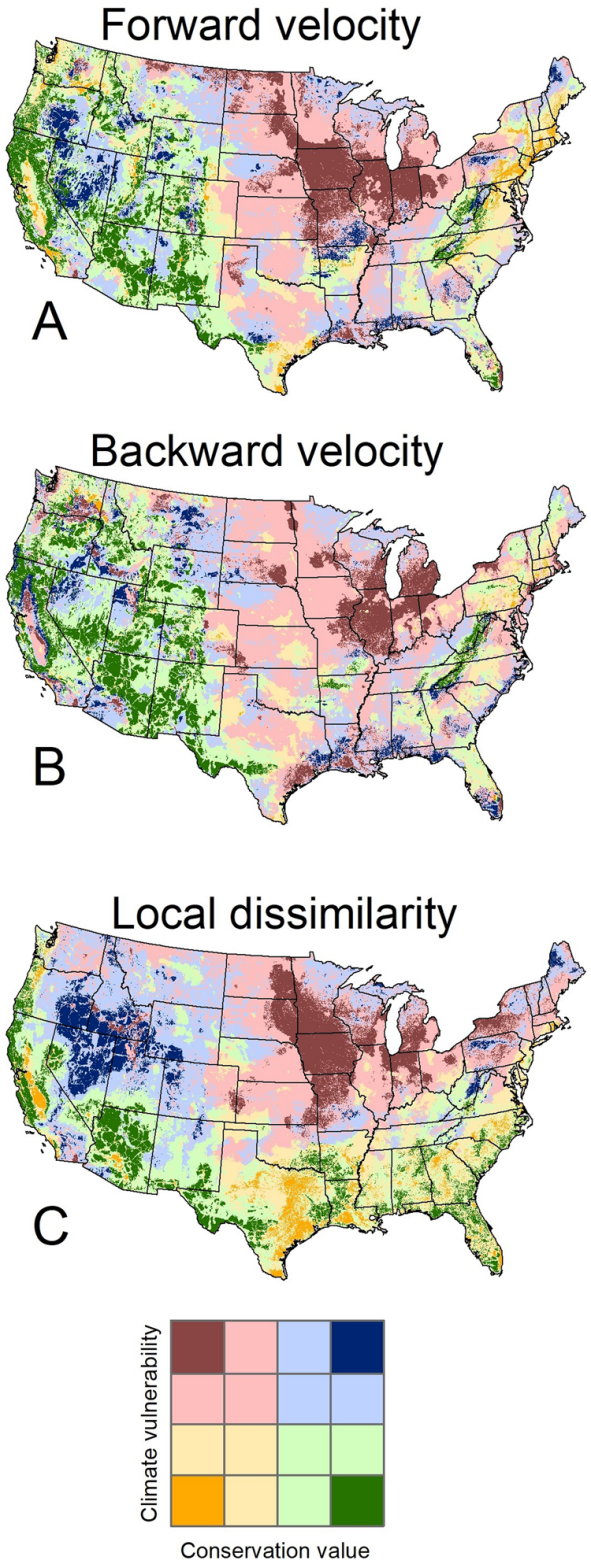
Bivariate maps of conservation value and climate vulnerability using three climate metrics: forward velocity (**A**), backward velocity (**B**), and local climate dissimilarity (**C**).

Variability in metrics resulted in different classification of vulnerability in the bivariate framework and maps (Fig. 4), although some broad patterns of similarity are evident in the maps. Land classified as low conservation value and low climate vulnerability was consistently less abundant than the other classifications among the three metrics (Table 1). Among the three metrics, lands classified as either low value-high vulnerability or high value-low vulnerability represented on average 36% and 37% of all lands, respectively.

**Table 1:** Percent area of the contiguous U.S. classified based on combinations of the degree of climate exposure (vulnerability) and conservation value for three multivariate metrics (i.e., the “corners” of the conceptual model and mapping legend).

	Forward velocity	Backward velocity	Climate dissimilarity
Low conservation value, low climate vulnerability	8.2	2.8	13.5
Low conservation value, high climate vulnerability	37.8	34.8	36.0
High conservation value, low climate vulnerability	37.2	45.9	29.5
High conservation value, high climate vulnerability	16.8	16.5	21.0

**Figure 5:**
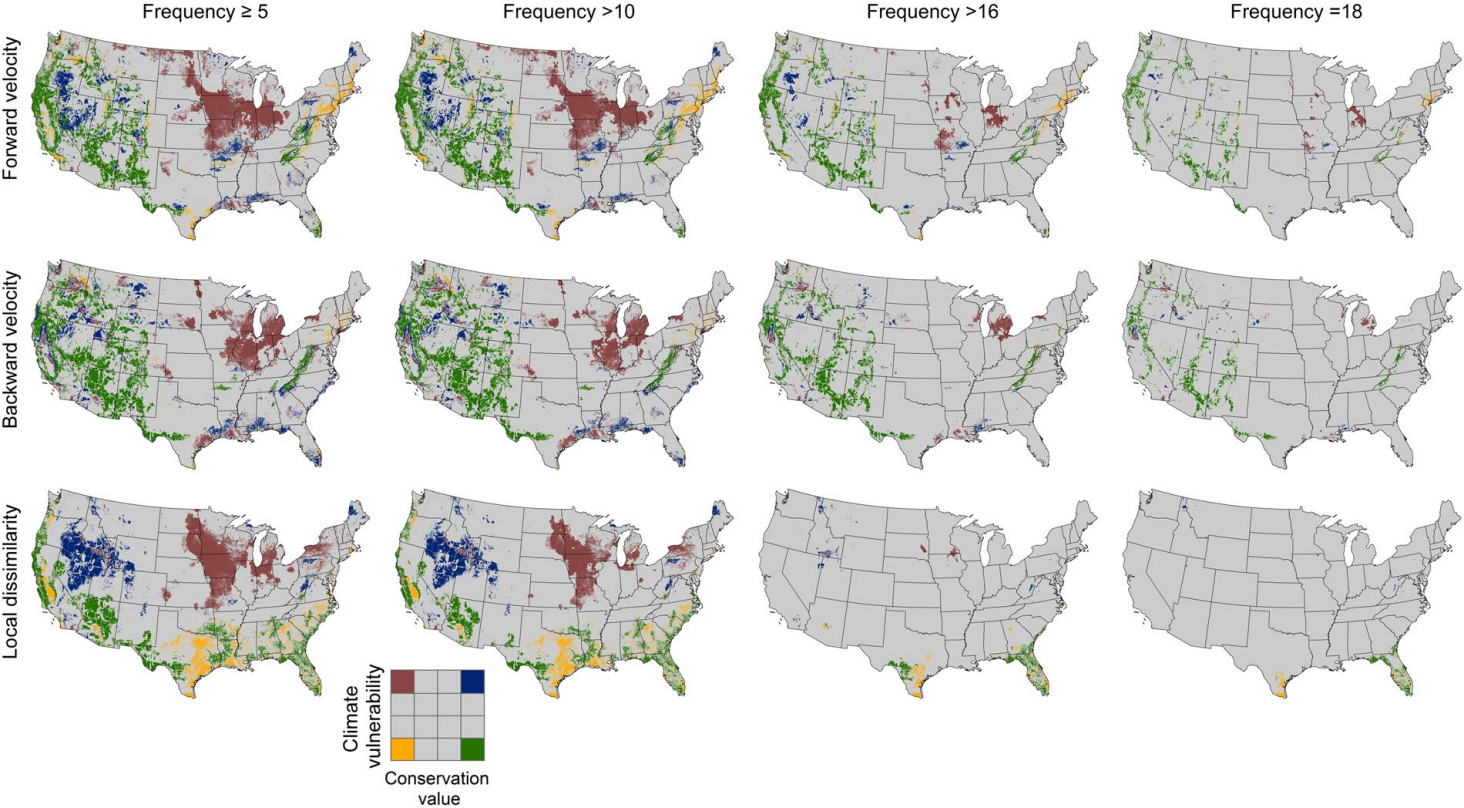
Maps of locations occupying the four corners of the conceptual framework of Belote *et al*.^11^, based on forward velocity (top row), backward velocity (middle row), and climate dissimilarity (bottom row), with columns showing the winnowing that happens with increasingly stringent thresholds for climate model consensus, ranging from low at left to high at right: ≥5 (left-hand column), >10 (second column from left), >16 (third column from left), or 18 (right-hand column). Area of each corner is shown in Fig. 6.

**Figure 6:**
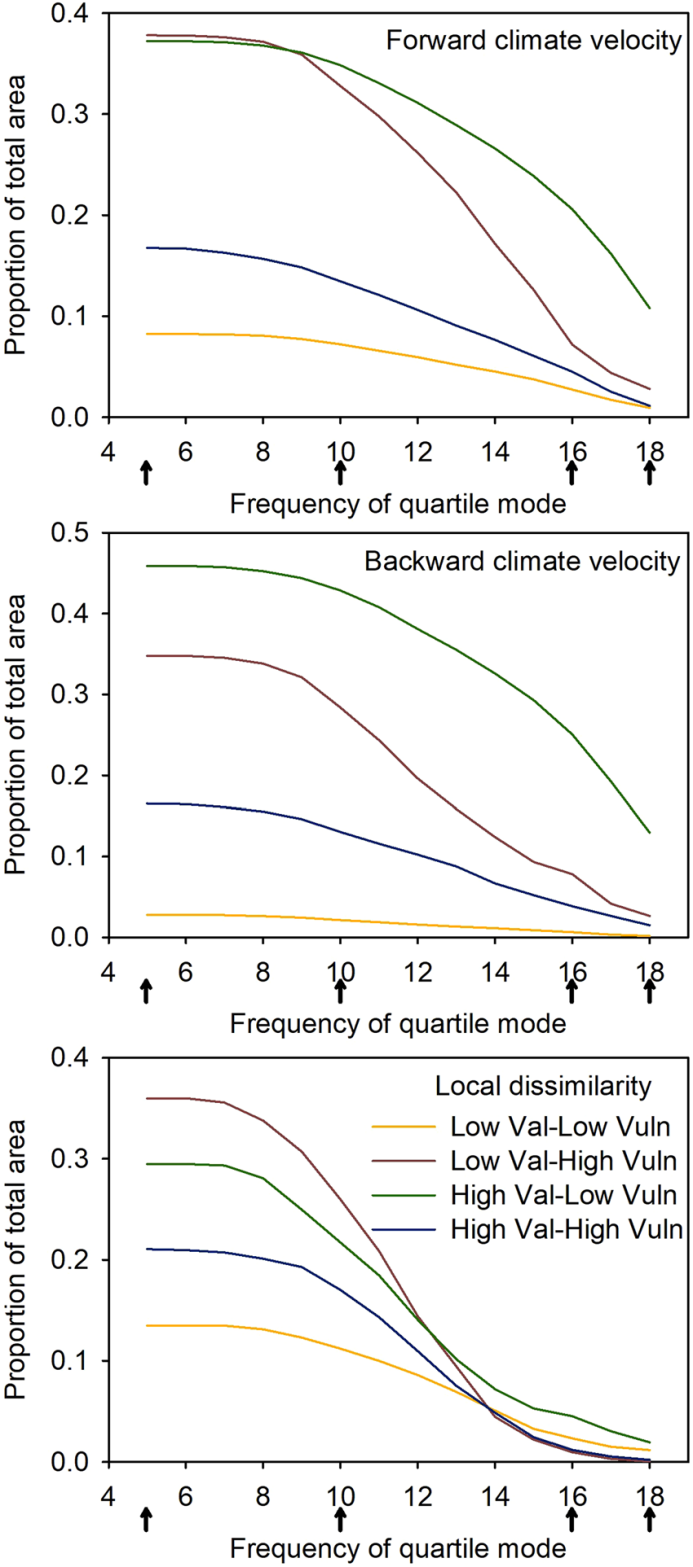
Change in area (represented as the proportion of total mapped area of the contiguous U.S.) for the four corners (i.e., Low or High conservation value × Low or High climate Vulnerability) along gradients in confidence as measured by the frequency of quartile mode. Arrows along x-axis represent conditions mapped in Fig. 5.

Lands are mapped in Fig. 4 and the left column of Fig. 5. Percent area is reported here for easier evaluation of geographic patterns in classification. Figure 6 shows how these values change when assessing agreement in climate simulations.

Setting increasingly high standards for model consensus resulted in decreasing areas where climate-informed conservation strategies could be confidently recommended (Figs 5 and 6). At the lowest threshold (5 out of 18 projections agree), large portions of the upper Midwestern states were classified as low value, high vulnerability for all three metrics. Similarly, many southwestern and western mountains were classified as high value, low vulnerability under all three metrics. As the standard of agreement was raised to 16, however, the area of agreement dropped markedly. Only metrics of velocity produced a high level of agreement for larger areas in the high value-low vulnerability classification, mainly in the mountainous regions of the West and in the Southern Appalachians. In contrast, climate dissimilarity showed high agreement only in Florida and central Texas.

## Discussion

Conservation planning increasingly uses climate predictions to inform allocation of strategies^10,14,16,17,55–57^. Our results confirm previous reports suggesting contrasting geographic patterns among different metrics of climate exposure, though we found a relatively high degree of agreement among the two metrics based on climate velocity metrics. Even though most regions were characterized by lack of full consensus in agreement among 18 climate projections for each exposure metric, several regions show relatively high degree of agreement in predictions for confidently assigning conservation strategies based on forward and backward velocity.

Areas where all three metrics (forward velocity, backward velocity, and climate dissimilarity) are either low or high were limited to small portions of the country, though at least two of the three metrics did align over relatively large areas. Mountainous regions throughout the West, for example, were characterized by low degrees of exposure in at least two metrics. These patterns are explained by similarities in the two velocity metrics, which are partially driven by topography. Future climate analogues may be in close geographic proximity to existing climate conditions in mountainous regions where steep elevation-driven climate gradients exist^25,26^. In contrast, areas in the upper Midwest and Great Basin regions were characterized by a high degree of predicted climate exposure for at least two of the three metrics. These areas with gentler topography or with greater predicted shifts in climate variables may be more vulnerable to greater ecological shifts (but see^58–60^).

Together, these geographic patterns of agreement among exposure metrics are consistent with previous assessments of multiple aspects of climate change and suggest that different exposure metrics vary in their geographic patterns^22,26^. Lack of alignment between these three metrics of climate exposure does not represent uncertainty per se, because the metrics are designed to measure different aspects of climate exposure. In fact, species and ecosystems of an area may experience different types of climate vulnerability based on different exposure metrics resulting in diverse responses to expected changes in climate^23^. However, it is important to acknowledge geographic variability in different multivariate indices of climate exposure^22,38^. High velocity represents exposure in terms of required movement rates for species persistence and colonization of ecosystems by climate-driven migrants^26^, whereas multivariate dissimilarity^28^ provides insights into local changes in climatic regimes, including shifts toward novel climate space^29,61^. Additional research should focus on the potential contrasting consequences of climate velocity and local multivariate shifts on species and ecosystems.

Our results provide insights for assigning conservation strategies based on projected future climate exposure. For example, conservation strategies aimed at anticipating climate and using innovative techniques (upper left corner of the conceptual framework; dark red mapped areas) may be more confidently applied to various lands located in the upper Midwest where the frequency in agreement of climate projections is relatively high ( ≥ 16 of 18, or ~89% of projections), at least for velocity metrics. These lands experience a high degree of human modification^50^, and restoration of lands within the matrix of agricultural lands may provide opportunities to emphasize innovative activities that anticipate the relatively high degree of climate exposure. While many of these lands are privately held, the return on the investment for conservation of species may be relatively high^62^.

Lands of high conservation value and high agreement among climate simulations and metrics of the mountainous West and Appalachian East could most confidently be assigned to conservation strategies aimed at traditional ecological reserve designations^17^. These lands represent the lower right-hand corner of the conceptual framework (green mapped areas) and were characterized by relative high degree of agreement among alternative projections, including several areas with full consensus for both velocity estimates.

The dryland regions of southeastern Oregon, northern Nevada, and parts of southern Idaho all represent high value-high vulnerability lands with at least some areas from each metric receiving a relatively high level of agreement among models (upper right region of the conceptual heuristic; blue mapped areas). These lands are relatively remote, located between protected areas, and composed of ecosystems under-represented in existing conservation reserves^12^. The relatively high degree of climate exposure expected in these areas presents conservation challenges. Calls to intervene through land management directed at facilitating ecosystem change in areas of high climate vulnerability are often countered by recommendations to protect climate vulnerable ecosystems^13^. Finding the best strategy to sustain biological diversity into the future in high value – high vulnerability lands is difficult. In these places, we recommend applying a range of conservation strategies that will spread risk and facilitate experimentation intended to maintain biodiversity and ecological processes into the future^63^. These strategies may range from maintaining or restoring conditions, managing for anticipating future conditions, and maintaining landscapes as untreated controls applied as experimental treatments^64,65^.

While several regions included lands frequently classified into the same bivariate value-vulnerability categories from the heuristic, full consensus among alternative climate projections was rare. Confidence in assignment of conservation strategies must take into account uncertainty and other important considerations. First, the different GCMs evaluated here represent alternative projections^37^, each of which may better predict certain aspects of climate regimes in certain geographic regions^66^. Our method of pooling GCM predictions does not consider the relative strengths of the GCMs in different regions. Second, the conservation values and climate vulnerability conceptual figure provides a heuristic framework to consider climate adaptation options. However, mapping areas based on this two-dimensional framework requires binning climate data to create classified bivariate value-vulnerability maps. Therefore, geographic regions of low climate exposure, for example, are based on values relative only to the distribution of data and the spatial scale represented. Thus, even lands of lower relative climate exposure may experience a high degree of change resulting in species- and ecosystem-altering effects that might require adjustments in conservation actions^67^.

Using binned quartiles of climate exposure estimates may conceal areas of agreement where predictions among different GCMs agree in the overall *direction* of climate change, but vary in predictions of magnitude. We provide predictions of change in the 11 climate variables used in the multivariate exposure metrics for all ecoregions^68^ and represent the variability among the 18 different GCM and RCP combinations as box and whisker plots (Supplemental Fig. 6). Summaries for the three multivariate exposure metrics are also reported to provide ranges of values for each metric. Ecoregions were used in these summaries to simplify climate predictions among defined geographic regions. These summaries of climate metric predictions among regions provide insights into the central tendency of magnitude and direction of climate predictions while representing the variability among simulation alternatives. For example, and not surprisingly, all projections predict an increase in temperatures of varying magnitudes, while precipitation is more variable. Some regions and models are expected to experience increases or decreases in precipitation. However, even in regions where precipitation is expected to increase, rising temperatures are predicted to increase estimates of moisture deficit. Evaluating the direction and magnitude of individual climate variables may provide important insights into sources of uncertainty among climate simulation predictions. It would be useful if such evaluations of individual climate variables accompanied work describing synthetic climate exposure metrics, such as those based on multivariate data reduction methods.

Additional sources of uncertainty include limited understanding of how species and ecological processes will respond to various aspects of climate change^22,32^. Responses of species and ecosystems to shifts in local climate regimes and displacement of climate analogues will likely be complex and could result in outcomes not reflected in our results^60,69^. For example, many mountainous regions were classified as relatively low exposure based on velocity metrics. However, it is important to note that species in mountainous areas may actually be more vulnerable than typically considered when accounting for potential exposure, if species must cross low elevation lands to reach a future climate analogue location^58^. Also, as climate analogues shift upslope their total area can contract^70^, which could also reduce ecological diversity within ecosystems dependent on those mountainous climate regimes^71^. Furthermore, we assumed that higher velocities and greater shifts in climate will be accompanied by a higher probability of resultant changes in species composition and ecological processes^22^, but this is not always the case^67^.

Other sources of uncertainty include which conservation strategies to emphasize in areas of intermediate values or vulnerability. The proposed heuristic framework and mapping approach of Belote *et al*.^17^ bring existing conservation values and estimates of climate vulnerability to bear on conservation planning or scenario development (*sensu*^30^). We emphasize the “corners” of the conceptual framework and mapping approach assuming conservation managers would be most confident when relatively high or low values and vulnerability occur. A general heuristic framework such as the one proposed by Belote *et al*.^17^ may be less useful when considering the middling areas. It is important to consider these caveats when evaluating maps based on the heuristic we present here, or when using data to map areas based on similar climate adaptation frameworks^15,16^.

Given our results and other considerations mentioned above, we offer four primary recommendations. First, decision-makers could use our approach to understand the central tendency of climate exposure metrics while explicitly evaluating confidence based our assessment of the frequency of assigning areas to high or low climate exposure. We provide an extension to the framework proposed by Belote *et al*.^17^ that incorporates uncertainty in climate predictions. Specifically, we recommend adjusting recommendations based on the level of certainty of climate exposure predictions (Table 2). However, confidence in decision making given levels of agreement in predictions of climate exposure will vary with the perceived or actual risks of management actions or inactions for various ecological conditions^63^ and conservation values such as habitat of sensitive species or ecosystems or hotspots of endemic species diversity^53^. Multiple lines of evidence should ultimately inform any conservation decision focused on adapting to expected climate change^72^.

**Table 2:** Framework for incorporating uncertainty associated with variability of climate predictions into conservation planning.

	Low conservation value		High conservation value	
	Low climate uncertainty	High climate uncertainty	Low climate uncertainty	High climate uncertainty
High climate vulnerability	Emphasize innovation	Emphasize innovation and prepare for surprises	Emphasize reserves and provide for flexibility	Emphasize reserves and plan for experimental approach
Low climate vulnerability	Emphasize restoration	Emphasize restoration and prepare for surprises	Emphasize traditional reserves	Emphasize reserves and provide for flexibility

Based on the framework of Belote *et al*.^17^ (Fig. 1), we adjust recommendations given certainty in predictions of climate exposure. In areas of low conservation value where restoration of active innovative management may be required to restore ecological functions and biodiversity, a high of uncertainty in climate exposure may lead to “surprises” in responses. In areas of high conservation values, where reserves may protect lands of high ecological integrity or importance, a high degree of climate uncertainty may require an acknowledgement of management flexibility or use of experimental approaches to adaptively maintain biodiversity and ecological function.

Second, methods such as distribution discounting^73^; (Supplemental Fig. 5) use variability among climate projections to discount estimates of climate exposure. In Supplemental Fig. 5, we present the discounted mean for each exposure metric using the standard deviation of estimates among alternative projections. Use of the standard deviation represents an alternative for explicitly accounting for variability among climate change projections. We recommend use of discounted metrics of exposure where variability among alternative climate projections are available.

Third, given high uncertainty because of lack of projection agreement or other sources, we recommend investing in ‘no regrets’ strategies of climate adaptation^9,17,74^. Protecting critical lands from development, mitigating human impacts, and controlling problematic invasive species represent a few examples of “no regrets” strategies (e.g.^9^). Fourth, diverse conservation and natural resource strategies should be simultaneously and experimentally^65^ evaluated in a risk-spreading portfolio^63^. Given uncertainty of ecological responses to climate change and management^75^, adopting only one approach to addressing predicted or ongoing changes in climate is not prudent. However, haphazardly applying various strategies to restore or manage land could lead to lost opportunities for learning or adaptation, or worse, could result in choosing a maladaptive strategy with potentially long-term consequences^63^. Finally, we recommend ecological research continue to investigate physiological responses of species and ecological processes to climate gradients, as well as investigation in the ability of species to disperse, establish, and persist in response to climate change^49^ while accounting for variability based on alternative predictions^32^.

## Conclusions

Our results provide explicit evaluation and maps of uncertainty among simulations. Considering this variability should be an important aspect of scenario planning^30^, and we recommend that planners apply one or more of the approaches we describe to ensure climate adaptation plans are robust to the uncertainty of future climate change impacts. Conceptual frameworks such as the one used here serve as a heuristic for considering conservation challenges in the face of climate change, but care must be taken not to oversimplify such challenges when mapping climate predictions to inform recommendations.

### Data availability

All data are publicly available from AdaptWest (adaptwest.databasin.org), including climate data and the wildland conservation value used in the analysis.

## Electronic supplementary material

SUPPLEMENTAL MATERIALS
