# Supplementary material for: Assessing agreement among alternative climate change projections to inform conservation recommendations in the contiguous United States
Source: Sci Rep. 2018 Jun 21;8:9441. doi: 10.1038/s41598-018-27721-6 (PMC6013454; doi:10.1038/s41598-018-27721-6)
Supplement: Supplementary file 1 — SUPPLEMENTAL MATERIALS [file 41598_2018_27721_MOESM1_ESM.pdf]

SUPPLEMENTAL MATERIALS

Assessing agreement among alternative climate change projections to inform conservation recommendations in the contiguous United States

R. Travis Belote, Carlos Carroll, Sebastián Martinuzzi, Julia Michalak, John W. Williams, Matthew A. Williamson, Gregory H. Aplet

Supplemental Table 1. CMIP5 general circulation models (GCMs) used to evaluate agreement among simulations. The first eight were used as individual simulations with RCP 4.5 and 8.5. In addition to a multi-model ensemble was calculated based on the first eight and the last seven GCMs listed.

Use	GCM	Source
Focal simulations	CanESM2	Canadian Centre for Climate, Canada
	CCSM4	National Center for Atmospheric Research, USA
	CNRM-CM5	Centre National de Recherches Meteorologiques, France
	GFDL-CM3	Geophysical Fluid Dynamics Laboratory, USA
	HadGEM2-ES	Met Office Hadley Centre, UK
	INM-CM4	Institute for Numerical Mathematics, Russia
	IPSL-CM5A-MR	Institute Pierre-Simon Laplace, France
	MPI-ESM-LR	Max Planck Institute for Meteorology, Germany
For Ensemble only	ACCESS1.0	Commonwealth Scientific and Industrial Research Organization, Australia
	MIROC5	Atmosphere and Ocean Research Institute, Japan
	CSIROMk3.6	Commonwealth Scientific and Industrial Research, Australia
	MRI-CGCM3	Meteorological Research Institute, Japan
	MIROC-ESM	Japan Agency for Marine-Earth Science and Technology, Japan
	CESM1-CAM5	National Center for Atmospheric Research, USA
	GISS-E2R	NASA Goddard Institute for Space Studies, USA

Supplemental Figure 1. Quartiles of all 18 alternative climate projections and scenario outputs for the forward climate velocity. Top nine map panels represent RCP 4.5; bottom 9 maps represent RCP 8.5.

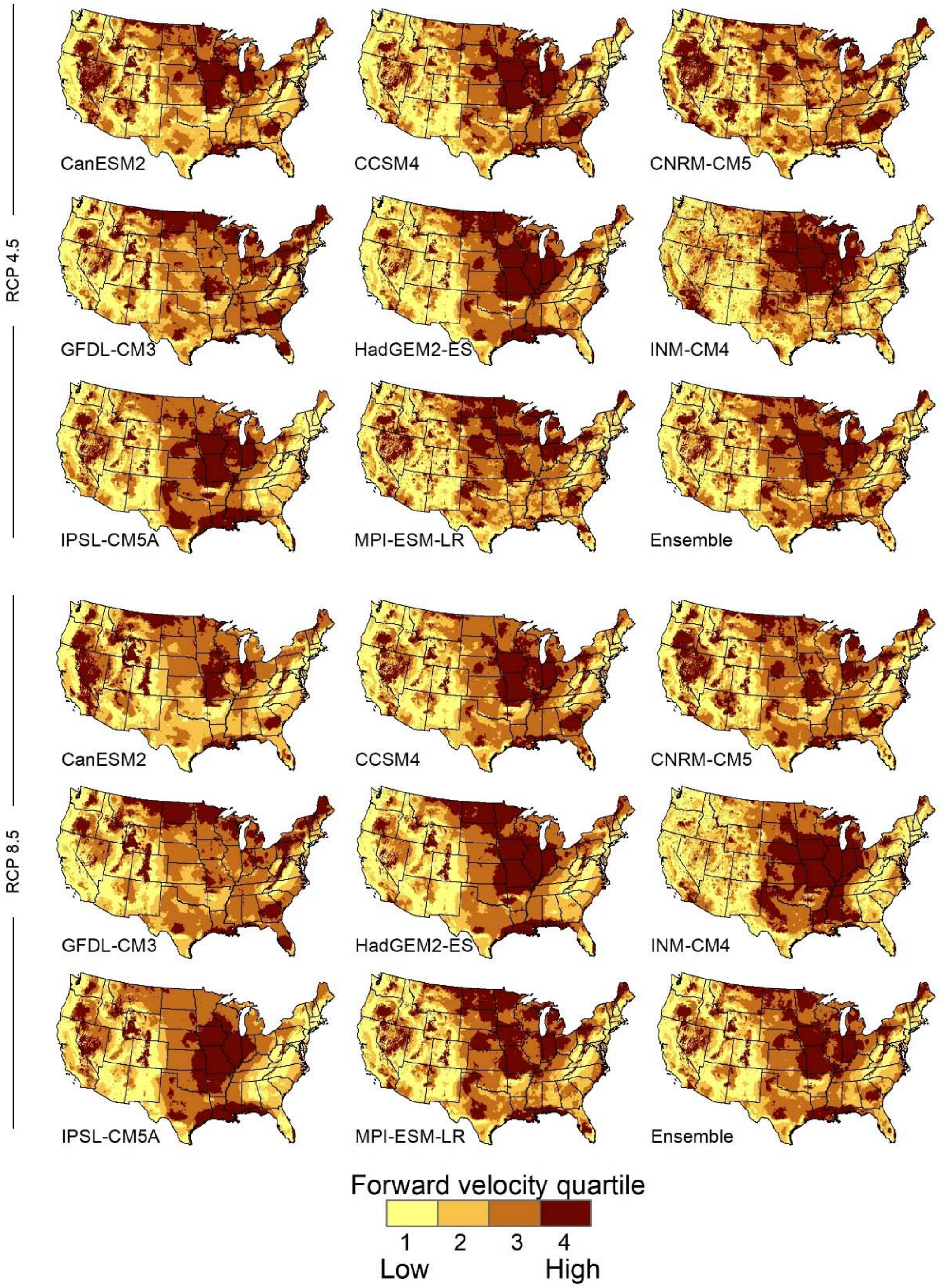

Supplemental Figure 2. Quartiles of all 18 alternative climate projections and scenario outputs for the backward climate velocity. Top nine map panels represent RCP 4.5; bottom 9 maps represent RCP 8.5.

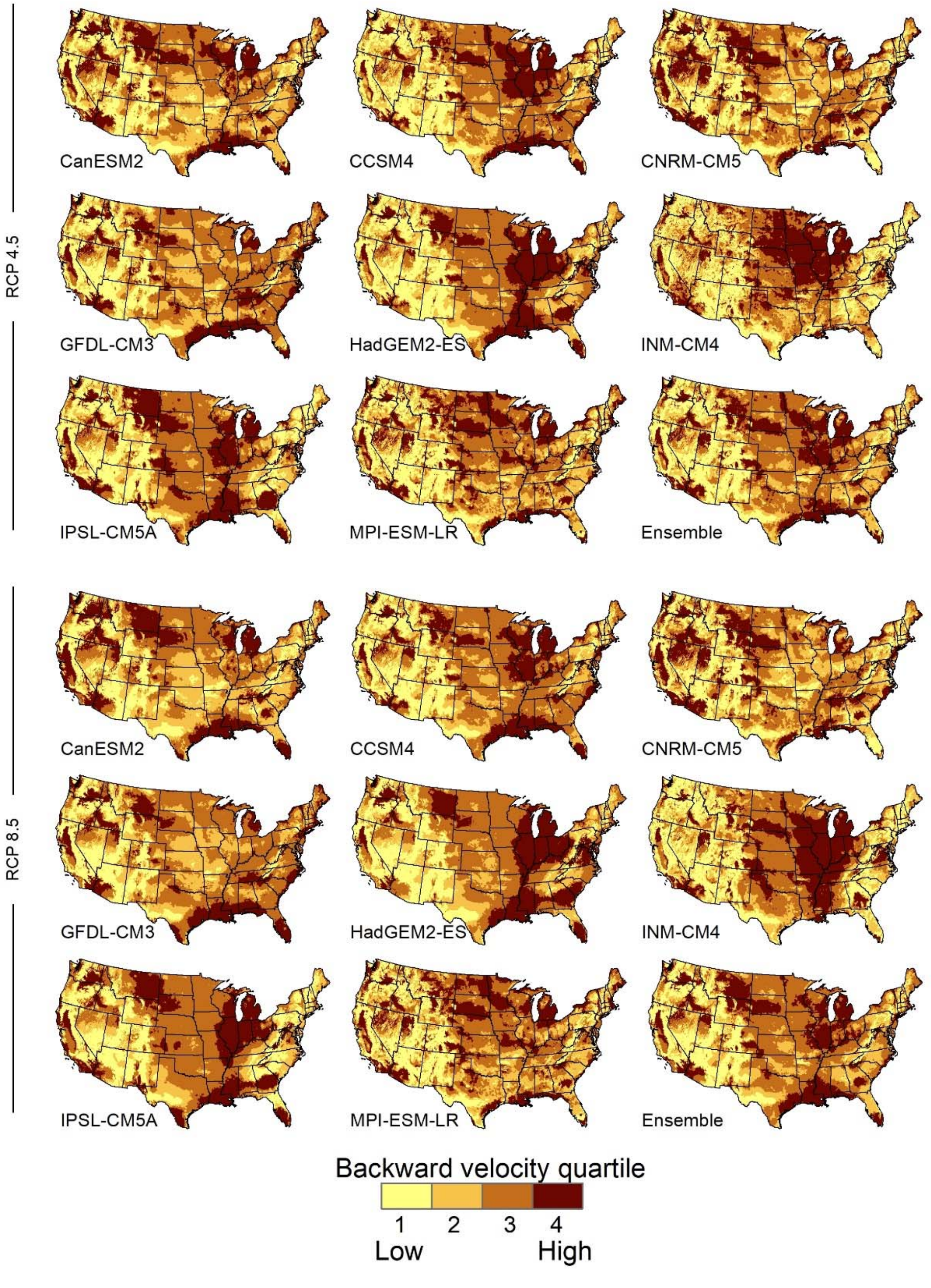

Supplemental Figure 3. Quartiles of all 18 alternative climate projections and scenario outputs for the local climate dissimilarity. Top nine map panels represent RCP 4.5; bottom 9 maps represent RCP 8.5.

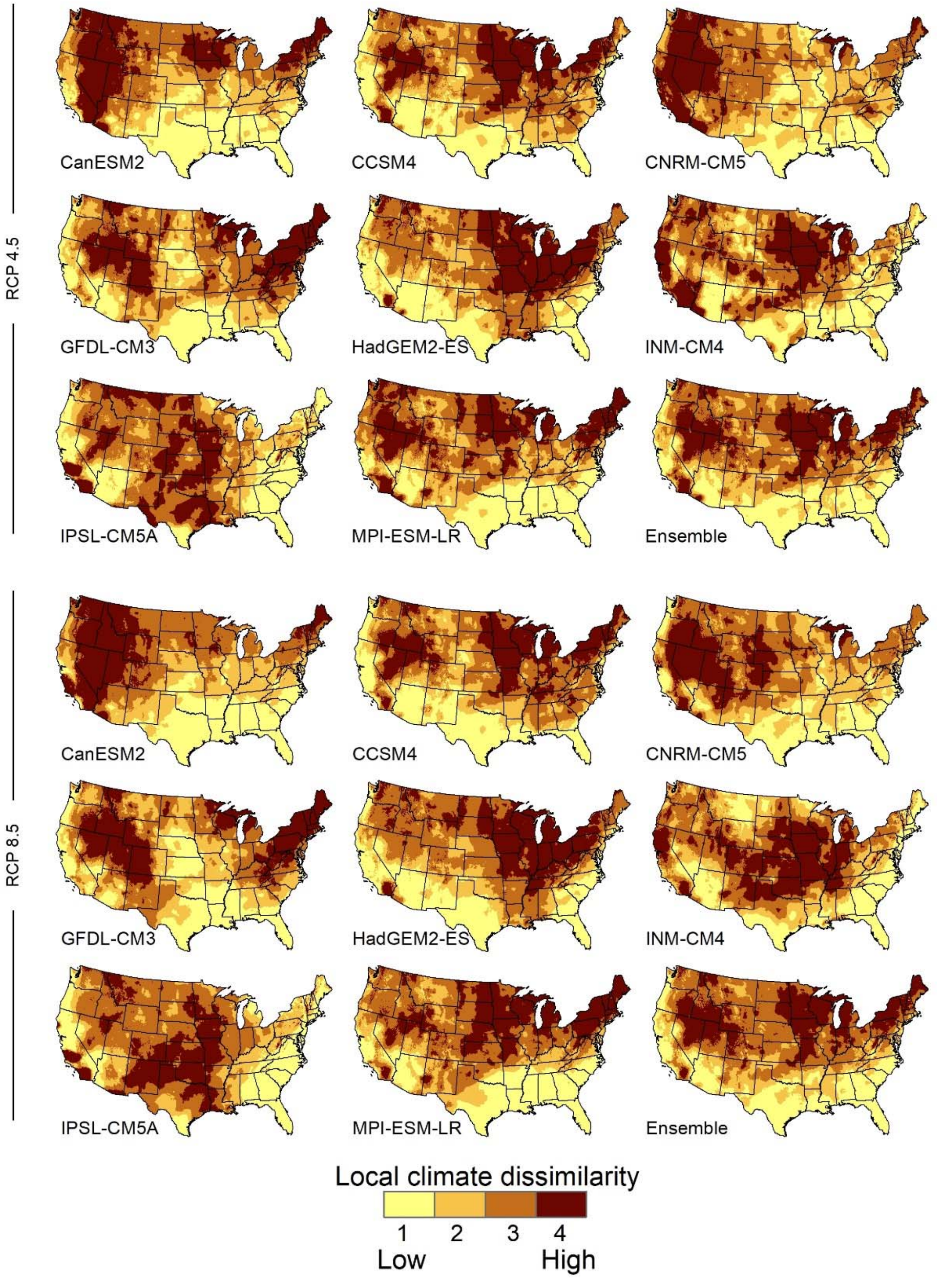

Supplemental Figure 4. Simplified depiction of methods used to assess simulation agreement. Each square represents one hypothetical example grid cell location. Three mapped climate metrics for the contiguous U.S. (forward velocity, backwards velocity, local climate dissimilarity) were classified into quartiles and assigned integer values 1 (lower quartile) to 4 (upper quartile). Within each climate exposure metric, classified quartile maps representing estimates from 18 different future climate simulations (9 general circulation models × 2 representative concentration pathways) were stacked. The mode of quartile and frequency of mode were calculated and used to produce maps of simulation agreement for each climate metric.

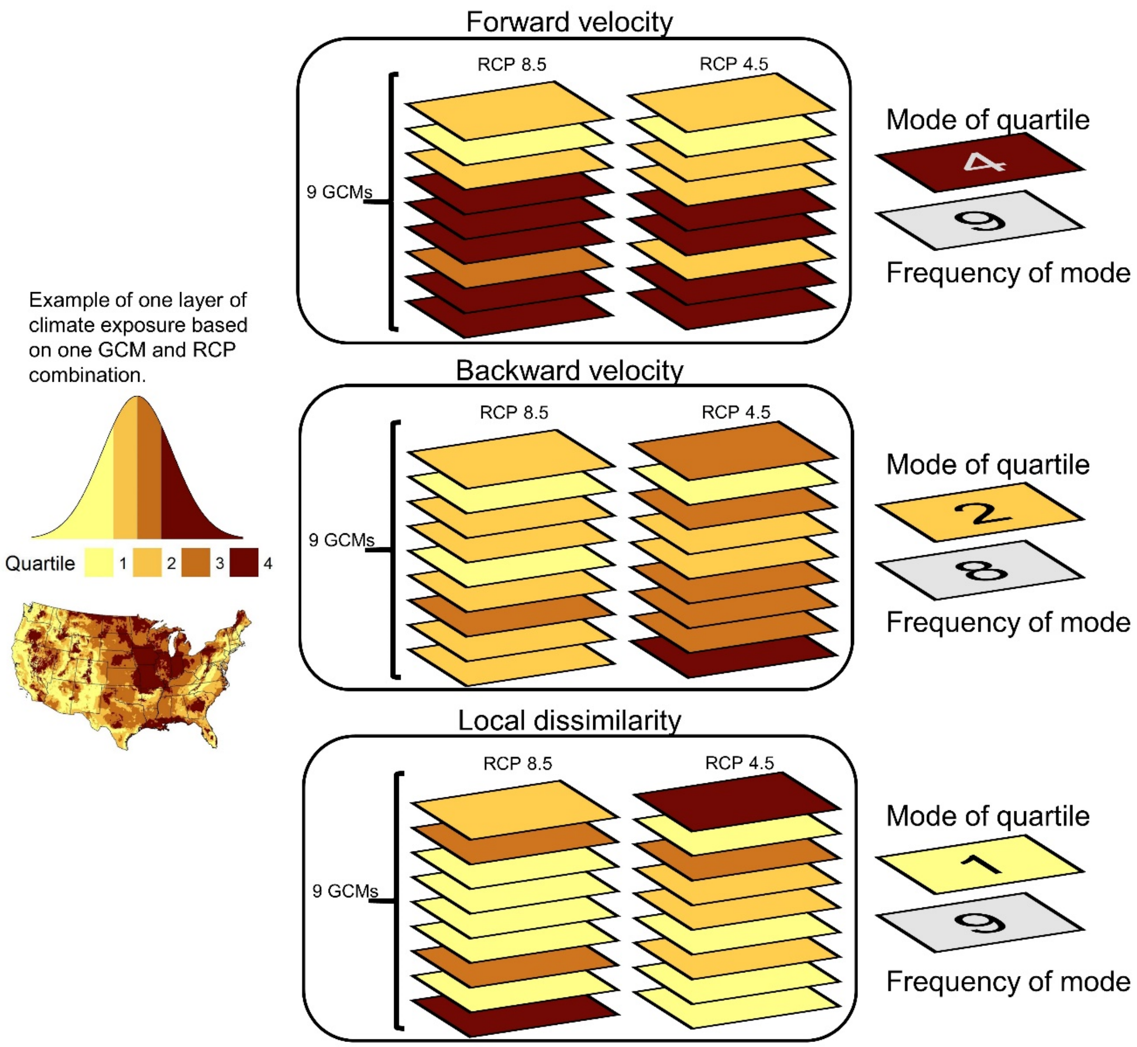

Supplemental Figure 5.

As a complement to the quadrant-based categorization described, we also applied an approach, distribution discounting, that reduces or discounts the estimated exposure of a site based on the level of uncertainty (e.g., the standard deviation between metrics based on alternative future climate projections) shown by metrics measured at that site[1,2]. Resultant conservation priority areas will then be located preferentially in areas of high agreement between alternative future projections. Because we were interested in assessing uncertainty of the three climate metrics when they were expressed as a conservation target (i.e., with positive values in areas of low climate exposure), we used a refugia index based on -1 times the log transform of the raw metrics[3]. We followed Carroll et al. (2010)[3] in discounting the mean value of the metric across the 18 future projections by one-half of the standard deviation of the metric between future projections. A range of discounting coefficients have been used (e.g., 1.0 rather than 0.5 as used here[2]), corresponding to differing levels of risk aversion.

Although regional patterns of uncertainty differed between the three metrics (with Pearson's correlations of 0.38-0.46), all metrics showed highest uncertainty in the southeastern US and intermountain West. Standard deviation was moderately correlated with mean values for climate dissimilarity (Pearson's correlation = 0.62) but not for either forward or backward velocity (Pearson's correlation = 0.18 and 0.25, respectively). The standard deviation and frequency of climate modes, two estimates of simulation agreement, were not strongly correlated for any metric (forward velocity: $r = -0.08$; backward velocity: $r = -0.15$; local dissimilarity: $r = -0.13$; although p-values were < 0.0001 for each correlation). Though areas with higher standard deviation among simulations were also characterized by lower frequency of climate modes.

Because the relative uncertainty of environmental dissimilarity values was correlated with mean values, use of distribution discounting spread priorities more broadly as compared to priorities based on undiscounted mean values. Patterns of uncertainty for forward and backward velocity were more complex, but generally lowered priorities in the intermountain West and southeastern US. Discounted means could be used as a substitute for the ensemble or median or mean values in bivariate mapping efforts.

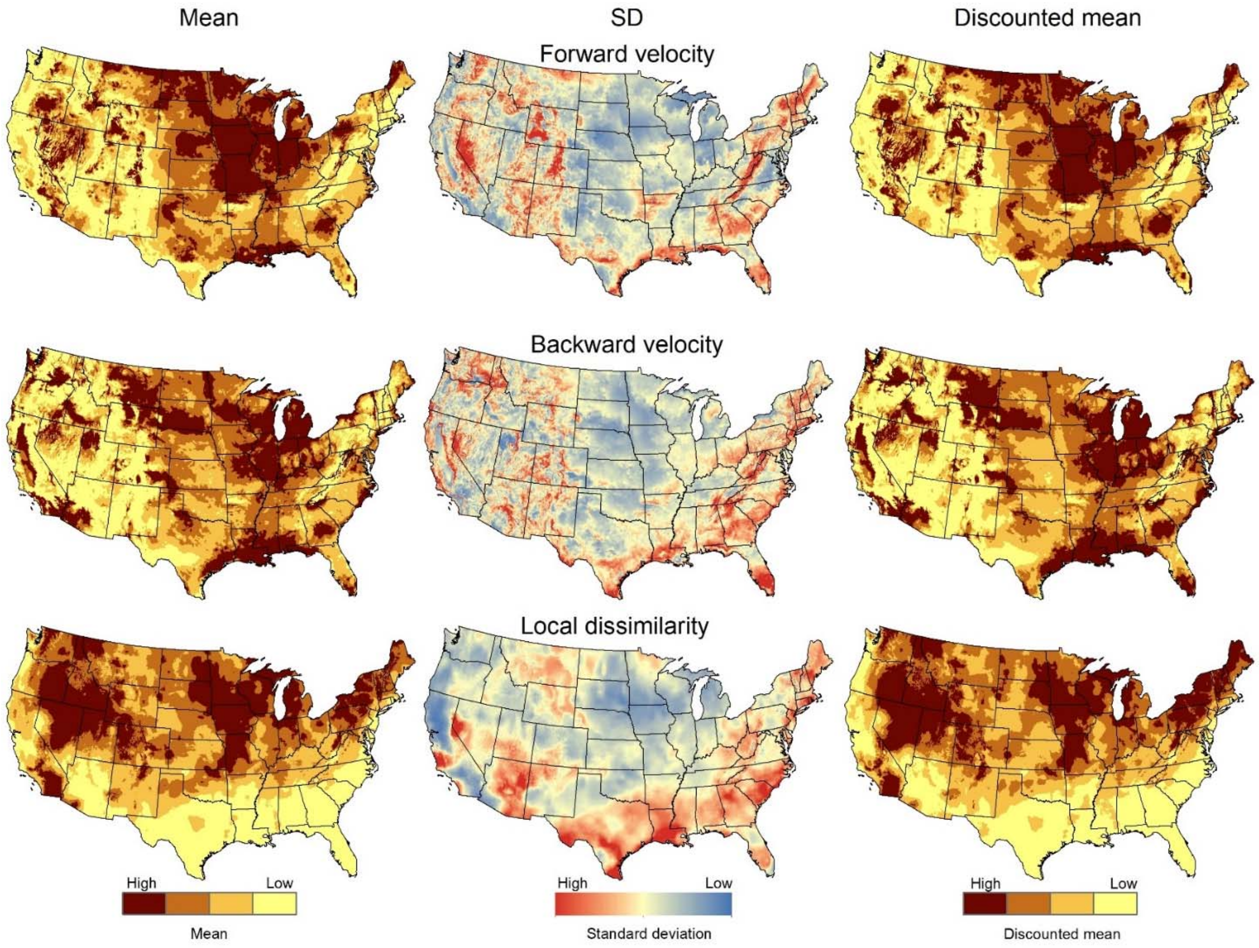

Supplemental Figure 6.

We summarized patterns of predicted climate change through the continuous US by EPA level 3 ecoregions[5]. We calculated the mean value of the three multivariate exposure metrics

(forward velocity, backward velocity, and local climate dissimilarity) for all 18 climate projections (based on the combinations of GCM and RCP alternatives). We also calculated the predicted degree of change in the 11 climate variables used in the principal components analysis (see list below) that served as the basis for calculating the multivariate exposure metrics. For temperature variables, we calculated the arithmetic difference between future predictions and current observations. For precipitation and number of day variables, we calculated the percent change between current and future values.

After calculating the mean values for each variable within each ecoregion, we plotted the data using box and whisker plots to summarize variability among ecoregions and alternative climate projections. The variability depicted by the box and whisker plots represent alternative predictions based on the 18 projections. We rank ordered these plots by the median value among the 18 simulation alternatives.

Code	Variable
MAT	mean annual temperature (°C)
MWMT	mean temperature of the warmest month (°C)
MCMT	mean temperature of the coldest month (°C)
TD	difference between MCMT and MWMT, as a measure of continentality (°C)
MAP	mean annual precipitation (mm)
MSP	mean summer (May to Sep) precipitation (mm)
MWP	mean winter (Oct to Apr) precipitation (mm)
DD5	degree-days above 5°C (growing degree days)
NFFD	the number of frost-free days
Eref	Hargreave's reference evaporation
CMD	Hargreave's climatic moisture index.

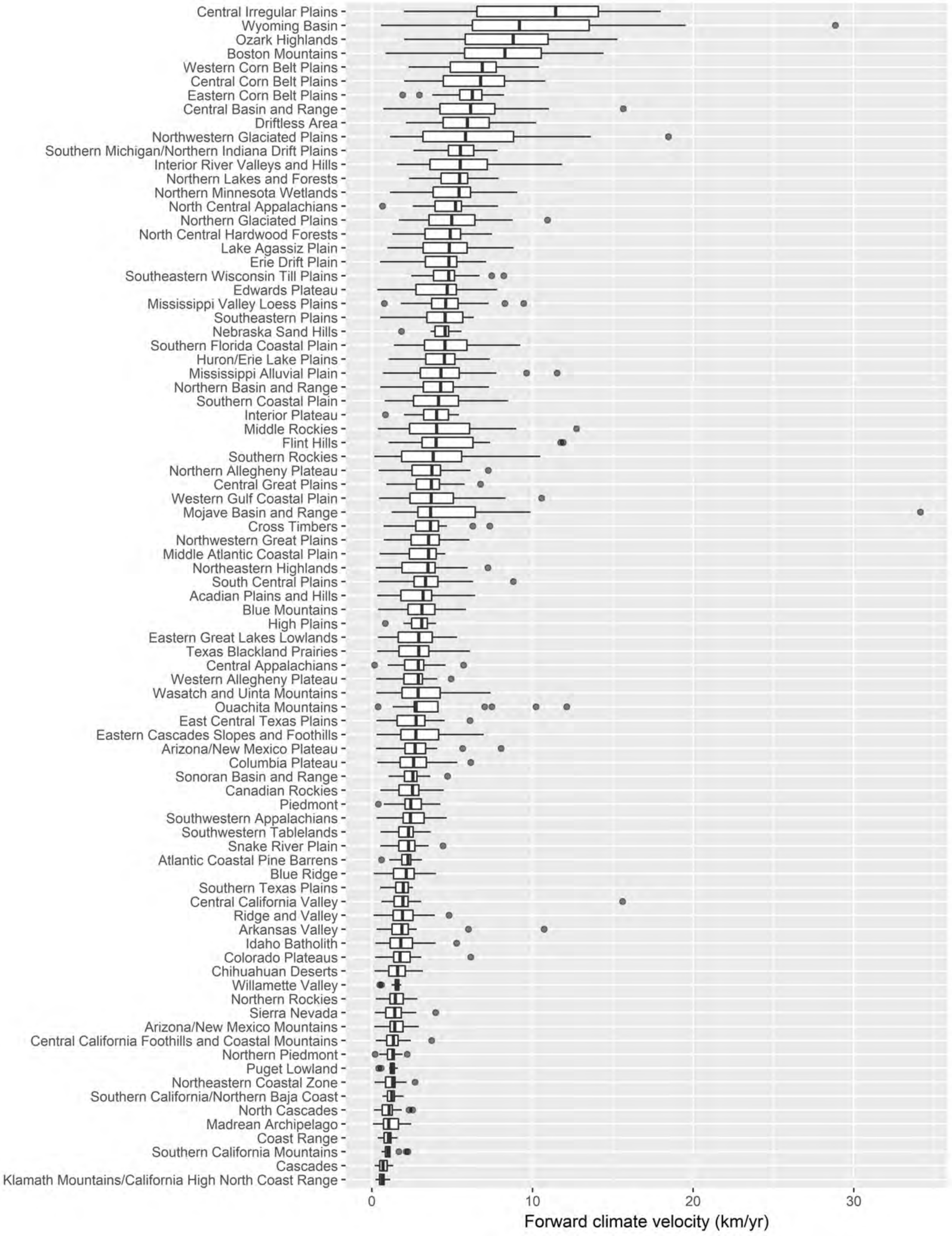

Central Irregular Plains
Wyoming Basin
Ozark Highlands
Boston Mountains
Western Corn Belt Plains
Central Corn Belt Plains
Eastern Corn Belt Plains
Central Basin and Range
Driftless Area
Northwestern Glaciated Plains
Southern Michigan/Northern Indiana Drift Plains
Interior River Valleys and Hills
Northern Lakes and Forests
Northern Minnesota Wetlands
North Central Appalachians
Northern Glaciated Plains
North Central Hardwood Forests
Lake Agassiz Plain
Erie Drift Plain
Southeastern Wisconsin Till Plains
Edwards Plateau
Mississippi Valley Loess Plains
Southeastern Plains
Nebraska Sand Hills
Southern Florida Coastal Plain
Huron/Erie Lake Plains
Mississippi Alluvial Plain
Northern Basin and Range
Southern Coastal Plain
Interior Plateau
Middle Rockies
Flint Hills
Southern Rockies
Northern Allegheny Plateau
Central Great Plains
Western Gulf Coastal Plain
Mojave Basin and Range
Cross Timbers
Northwestern Great Plains
Middle Atlantic Coastal Plain
Northeastern Highlands
South Central Plains
Acadian Plains and Hills
Blue Mountains
High Plains
Eastern Great Lakes Lowlands
Texas Blackland Prairies
Central Appalachians
Western Allegheny Plateau
Wasatch and Uinta Mountains
Ouachita Mountains
East Central Texas Plains
Eastern Cascades Slopes and Foothills
Arizona/New Mexico Plateau
Columbia Plateau
Sonoran Basin and Range
Canadian Rockies
Piedmont
Southwestern Appalachians
Southwestern Tablelands
Snake River Plain
Atlantic Coastal Pine Barrens
Blue Ridge
Southern Texas Plains
Central California Valley
Ridge and Valley
Arkansas Valley
Idaho Batholith
Colorado Plateaus
Chihuahuan Deserts
Willamette Valley
Northern Rockies
Sierra Nevada
Arizona/New Mexico Mountains
Central California Foothills and Coastal Mountains
Northern Piedmont
Puget Lowland
Northeastern Coastal Zone
Southern California/Northern Baja Coast
North Cascades
Madrean Archipelago
Coast Range
Southern California Mountains
Cascades
Klamath Mountains/California High North Coast Range
0
10
20
30
Forward climate velocity (km/yr)

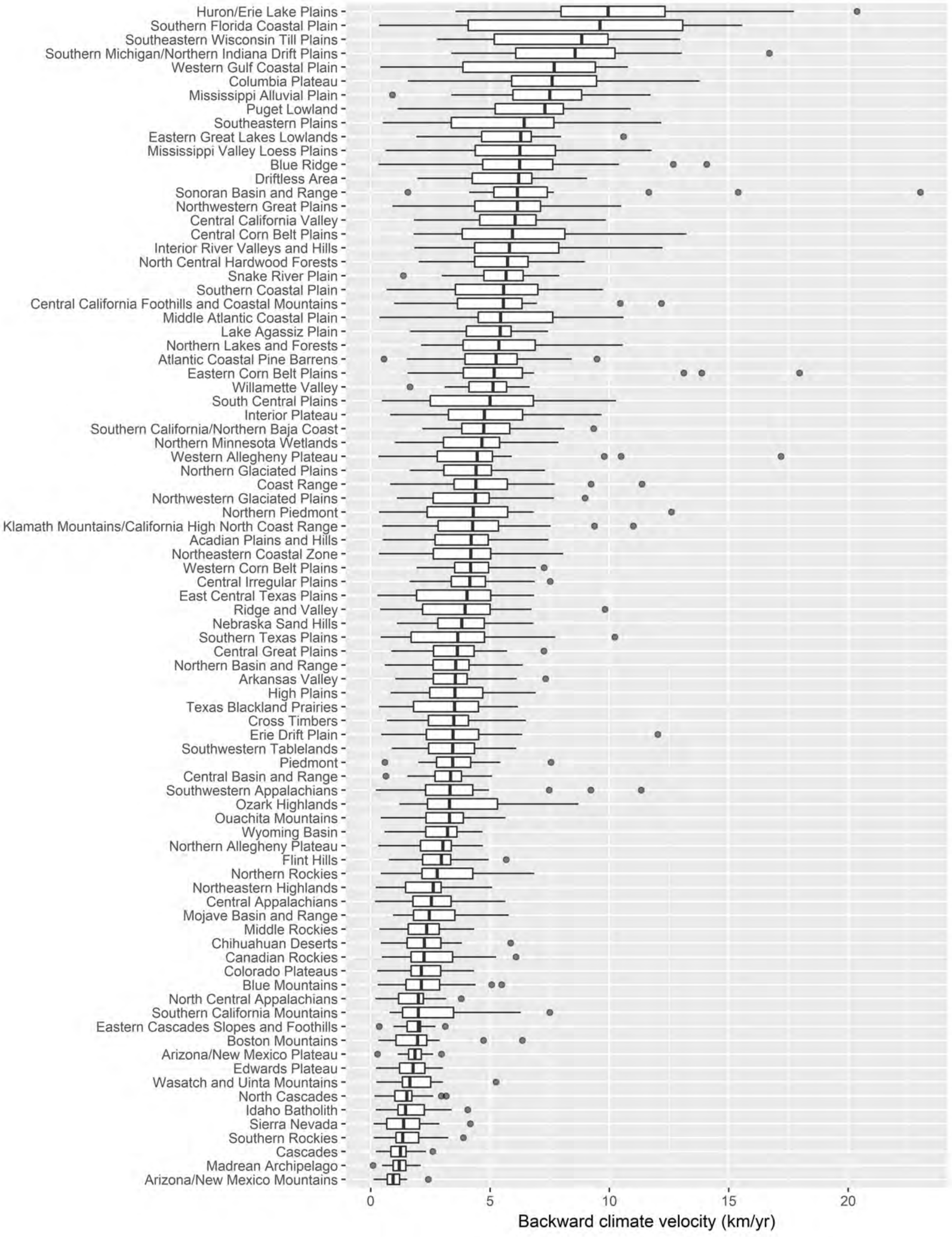

Huron/Erie Lake Plains
Southern Florida Coastal Plain
Southeastern Wisconsin Till Plains
Southern Michigan/Northern Indiana Drift Plains
Western Gulf Coastal Plain
Columbia Plateau
Mississippi Alluvial Plain
Puget Lowland
Southeastern Plains
Eastern Great Lakes Lowlands
Mississippi Valley Loess Plains
Blue Ridge
Driftless Area
Sonoran Basin and Range
Northwestern Great Plains
Central California Valley
Central Corn Belt Plains
Interior River Valleys and Hills
North Central Hardwood Forests
Snake River Plain
Southern Coastal Plain
Central California Foothills and Coastal Mountains
Middle Atlantic Coastal Plain
Lake Agassiz Plain
Northern Lakes and Forests
Atlantic Coastal Pine Barrens
Eastern Corn Belt Plains
Willamette Valley
South Central Plains
Interior Plateau
Southern California/Northern Baja Coast
Northern Minnesota Wetlands
Western Allegheny Plateau
Northern Glaciated Plains
Coast Range
Northwestern Glaciated Plains
Northern Piedmont
Klamath Mountains/California High North Coast Range
Acadian Plains and Hills
Northeastern Coastal Zone
Western Corn Belt Plains
Central Irregular Plains
East Central Texas Plains
Ridge and Valley
Nebraska Sand Hills
Southern Texas Plains
Central Great Plains
Northern Basin and Range
Arkansas Valley
High Plains
Texas Blackland Prairies
Cross Timbers
Erie Drift Plain
Southwestern Tablelands
Piedmont
Central Basin and Range
Southwestern Appalachians
Ozark Highlands
Ouachita Mountains
Wyoming Basin
Northern Allegheny Plateau
Flint Hills
Northern Rockies
Northeastern Highlands
Central Appalachians
Mojave Basin and Range
Middle Rockies
Chihuahuan Deserts
Canadian Rockies
Colorado Plateaus
Blue Mountains
North Central Appalachians
Southern California Mountains
Eastern Cascades Slopes and Foothills
Boston Mountains
Arizona/New Mexico Plateau
Edwards Plateau
Wasatch and Uinta Mountains
North Cascades
Idaho Batholith
Sierra Nevada
Southern Rockies
Cascades
Madrean Archipelago
Arizona/New Mexico Mountains
0
5
10
15
20
Backward climate velocity (km/yr)

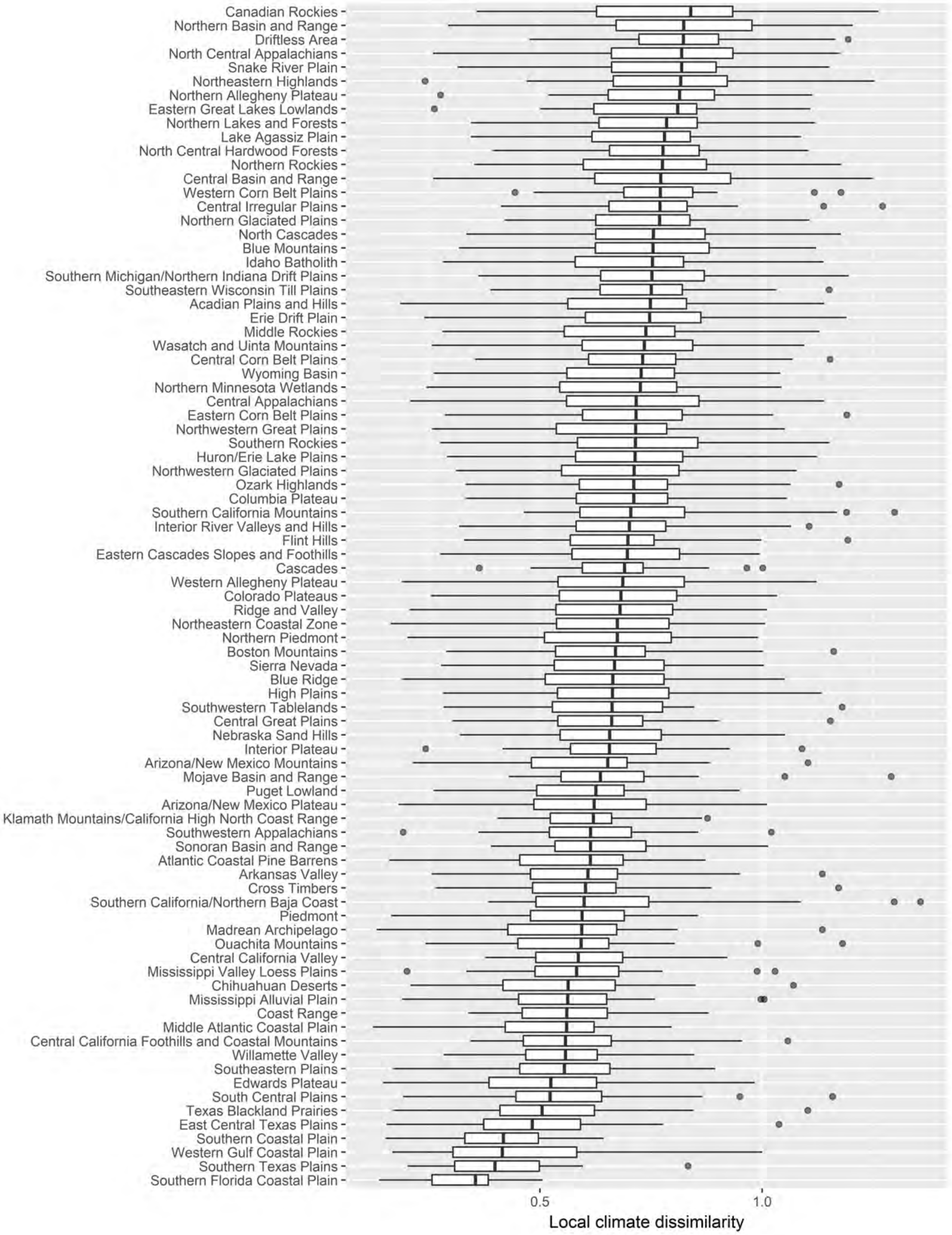

Canadian Rockies
Northern Basin and Range
Driftless Area
North Central Appalachians
Snake River Plain
Northeastern Highlands
Northern Allegheny Plateau
Eastern Great Lakes Lowlands
Northern Lakes and Forests
Lake Agassiz Plain
North Central Hardwood Forests
Northern Rockies
Central Basin and Range
Western Corn Belt Plains
Central Irregular Plains
Northern Glaciated Plains
North Cascades
Blue Mountains
Idaho Batholith
Southern Michigan/Northern Indiana Drift Plains
Southeastern Wisconsin Till Plains
Acadian Plains and Hills
Erie Drift Plain
Middle Rockies
Wasatch and Uinta Mountains
Central Corn Belt Plains
Wyoming Basin
Northern Minnesota Wetlands
Central Appalachians
Eastern Corn Belt Plains
Northwestern Great Plains
Southern Rockies
Huron/Erie Lake Plains
Northwestern Glaciated Plains
Ozark Highlands
Columbia Plateau
Southern California Mountains
Interior River Valleys and Hills
Flint Hills
Eastern Cascades Slopes and Foothills
Cascades
Western Allegheny Plateau
Colorado Plateaus
Ridge and Valley
Northeastern Coastal Zone
Northern Piedmont
Boston Mountains
Sierra Nevada
Blue Ridge
High Plains
Southwestern Tablelands
Central Great Plains
Nebraska Sand Hills
Interior Plateau
Arizona/New Mexico Mountains
Mojave Basin and Range
Puget Lowland
Arizona/New Mexico Plateau
Klamath Mountains/California High North Coast Range
Southwestern Appalachians
Sonoran Basin and Range
Atlantic Coastal Pine Barrens
Arkansas Valley
Cross Timbers
Southern California/Northern Baja Coast
Piedmont
Madrean Archipelago
Ouachita Mountains
Central California Valley
Mississippi Valley Loess Plains
Chihuahuan Deserts
Mississippi Alluvial Plain
Coast Range
Middle Atlantic Coastal Plain
Central California Foothills and Coastal Mountains
Willamette Valley
Southeastern Plains
Edwards Plateau
South Central Plains
Texas Blackland Prairies
East Central Texas Plains
Southern Coastal Plain
Western Gulf Coastal Plain
Southern Texas Plains
Southern Florida Coastal Plain
0.5
1.0
Local climate dissimilarity

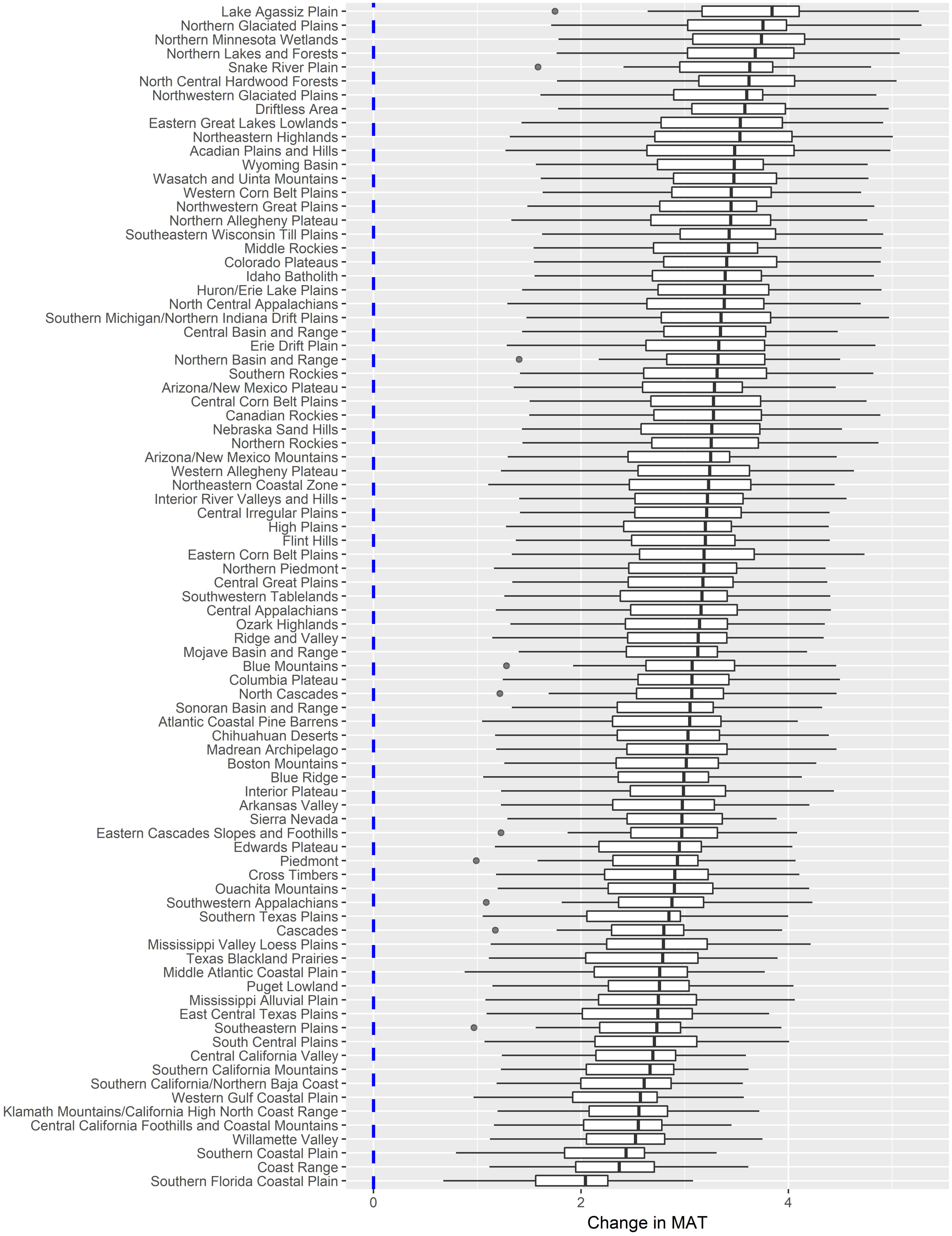

Lake Agassiz Plain
Northern Glaciated Plains
Northern Minnesota Wetlands
Northern Lakes and Forests
Snake River Plain
North Central Hardwood Forests
Northwestern Glaciated Plains
Driftless Area
Eastern Great Lakes Lowlands
Northeastern Highlands
Acadian Plains and Hills
Wyoming Basin
Wasatch and Uinta Mountains
Western Corn Belt Plains
Northwestern Great Plains
Northern Allegheny Plateau
Southeastern Wisconsin Till Plains
Middle Rockies
Colorado Plateaus
Idaho Batholith
Huron/Erie Lake Plains
North Central Appalachians
Southern Michigan/Northern Indiana Drift Plains
Central Basin and Range
Erie Drift Plain
Northern Basin and Range
Southern Rockies
Arizona/New Mexico Plateau
Central Corn Belt Plains
Canadian Rockies
Nebraska Sand Hills
Northern Rockies
Arizona/New Mexico Mountains
Western Allegheny Plateau
Northeastern Coastal Zone
Interior River Valleys and Hills
Central Irregular Plains
High Plains
Flint Hills
Eastern Corn Belt Plains
Northern Piedmont
Central Great Plains
Southwestern Tablelands
Central Appalachians
Ozark Highlands
Ridge and Valley
Mojave Basin and Range
Blue Mountains
Columbia Plateau
North Cascades
Sonoran Basin and Range
Atlantic Coastal Pine Barrens
Chihuahuan Deserts
Madrean Archipelago
Boston Mountains
Blue Ridge
Interior Plateau
Arkansas Valley
Sierra Nevada
Eastern Cascades Slopes and Foothills
Edwards Plateau
Piedmont
Cross Timbers
Ouachita Mountains
Southwestern Appalachians
Southern Texas Plains
Cascades
Mississippi Valley Loess Plains
Texas Blackland Prairies
Middle Atlantic Coastal Plain
Puget Lowland
Mississippi Alluvial Plain
East Central Texas Plains
Southeastern Plains
South Central Plains
Central California Valley
Southern California Mountains
Southern California/Northern Baja Coast
Western Gulf Coastal Plain
Klamath Mountains/California High North Coast Range
Central California Foothills and Coastal Mountains
Willamette Valley
Southern Coastal Plain
Coast Range
Southern Florida Coastal Plain
0
2
4
Change in MAT

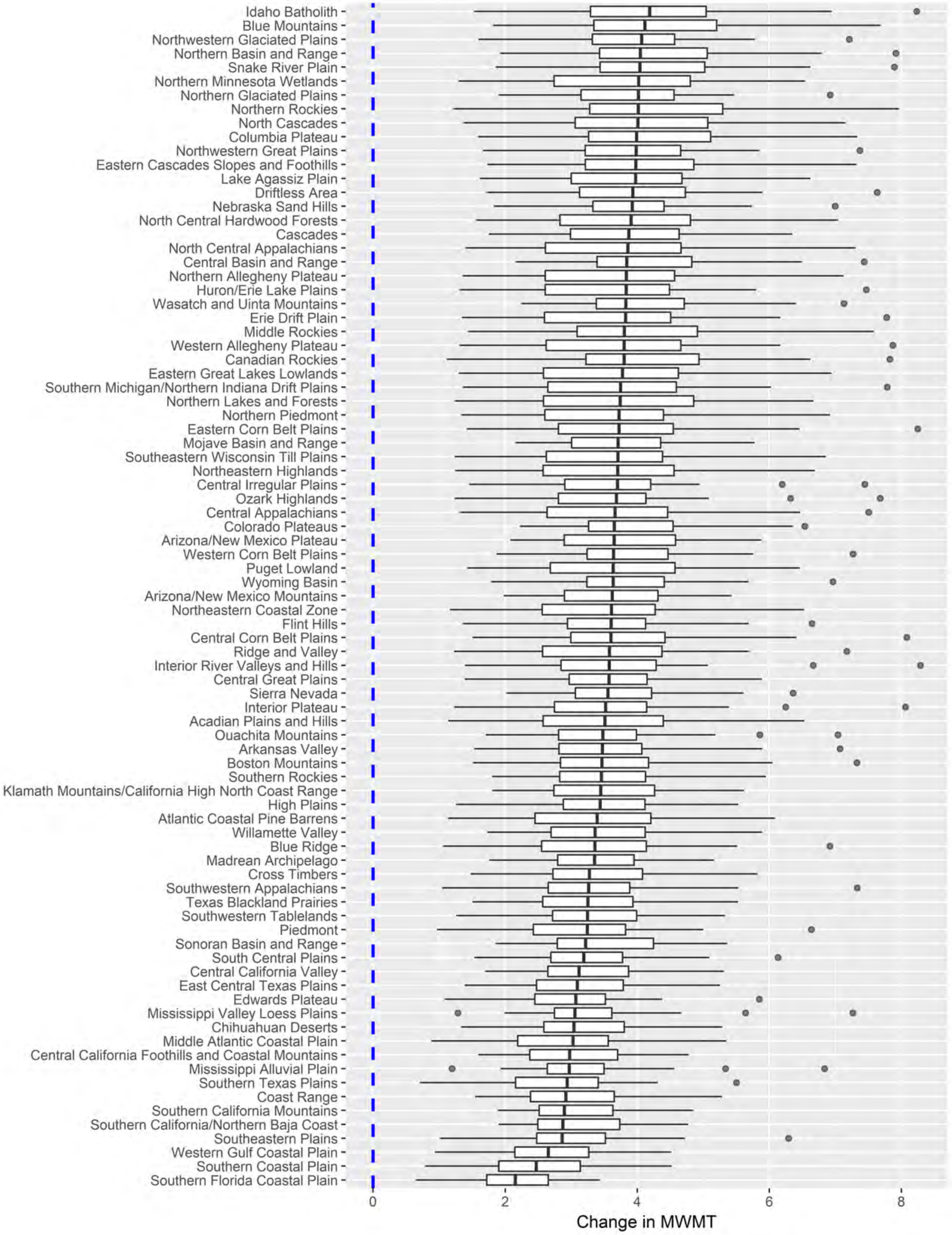

Idaho Batholith
Blue Mountains
Northwestern Glaciated Plains
Northern Basin and Range
Snake River Plain
Northern Minnesota Wetlands
Northern Glaciated Plains
Northern Rockies
North Cascades
Columbia Plateau
Northwestern Great Plains
Eastern Cascades Slopes and Foothills
Lake Agassiz Plain
Driftless Area
Nebraska Sand Hills
North Central Hardwood Forests
Cascades
North Central Appalachians
Central Basin and Range
Northern Allegheny Plateau
Huron/Erie Lake Plains
Wasatch and Uinta Mountains
Erie Drift Plain
Middle Rockies
Western Allegheny Plateau
Canadian Rockies
Eastern Great Lakes Lowlands
Southern Michigan/Northern Indiana Drift Plains
Northern Lakes and Forests
Northern Piedmont
Eastern Corn Belt Plains
Mojave Basin and Range
Southeastern Wisconsin Till Plains
Northeastern Highlands
Central Irregular Plains
Ozark Highlands
Central Appalachians
Colorado Plateaus
Arizona/New Mexico Plateau
Western Corn Belt Plains
Puget Lowland
Wyoming Basin
Arizona/New Mexico Mountains
Northeastern Coastal Zone
Flint Hills
Central Corn Belt Plains
Ridge and Valley
Interior River Valleys and Hills
Central Great Plains
Sierra Nevada
Interior Plateau
Acadian Plains and Hills
Ouachita Mountains
Arkansas Valley
Boston Mountains
Southern Rockies
Klamath Mountains/California High North Coast Range
High Plains
Atlantic Coastal Pine Barrens
Willamette Valley
Blue Ridge
Madrean Archipelago
Cross Timbers
Southwestern Appalachians
Texas Blackland Prairies
Southwestern Tablelands
Piedmont
Sonoran Basin and Range
South Central Plains
Central California Valley
East Central Texas Plains
Edwards Plateau
Mississippi Valley Loess Plains
Chihuahuan Deserts
Middle Atlantic Coastal Plain
Central California Foothills and Coastal Mountains
Mississippi Alluvial Plain
Southern Texas Plains
Coast Range
Southern California Mountains
Southern California/Northern Baja Coast
Southeastern Plains
Western Gulf Coastal Plain
Southern Coastal Plain
Southern Florida Coastal Plain
0
2
4
6
8
Change in MWMT

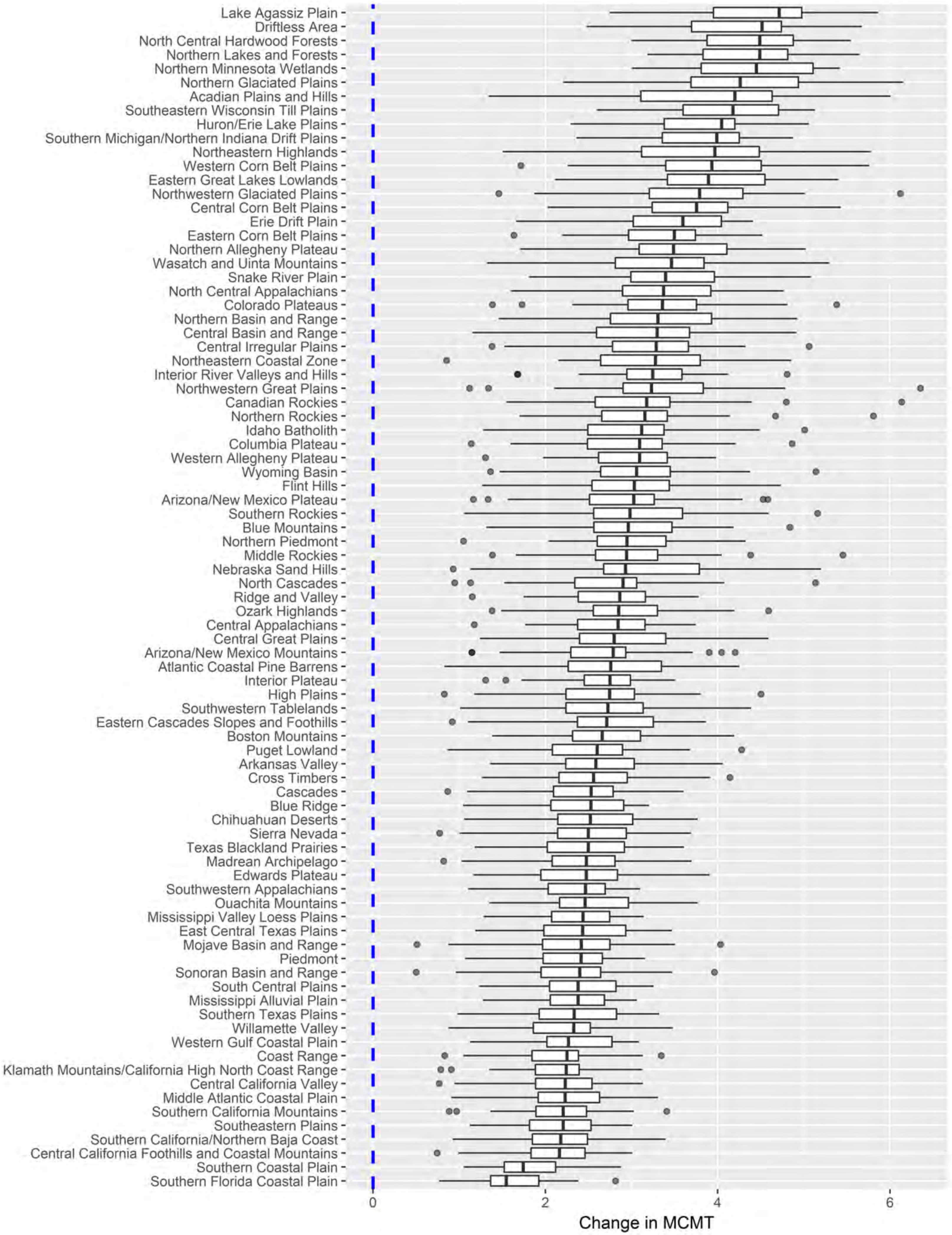

Lake Agassiz Plain
Driftless Area
North Central Hardwood Forests
Northern Lakes and Forests
Northern Minnesota Wetlands
Northern Glaciated Plains
Acadian Plains and Hills
Southeastern Wisconsin Till Plains
Huron/Erie Lake Plains
Southern Michigan/Northern Indiana Drift Plains
Northeastern Highlands
Western Corn Belt Plains
Eastern Great Lakes Lowlands
Northwestern Glaciated Plains
Central Corn Belt Plains
Erie Drift Plain
Eastern Corn Belt Plains
Northern Allegheny Plateau
Wasatch and Uinta Mountains
Snake River Plain
North Central Appalachians
Colorado Plateaus
Northern Basin and Range
Central Basin and Range
Central Irregular Plains
Northeastern Coastal Zone
Interior River Valleys and Hills
Northwestern Great Plains
Canadian Rockies
Northern Rockies
Idaho Batholith
Columbia Plateau
Western Allegheny Plateau
Wyoming Basin
Flint Hills
Arizona/New Mexico Plateau
Southern Rockies
Blue Mountains
Northern Piedmont
Middle Rockies
Nebraska Sand Hills
North Cascades
Ridge and Valley
Ozark Highlands
Central Appalachians
Central Great Plains
Arizona/New Mexico Mountains
Atlantic Coastal Pine Barrens
Interior Plateau
High Plains
Southwestern Tablelands
Eastern Cascades Slopes and Foothills
Boston Mountains
Puget Lowland
Arkansas Valley
Cross Timbers
Cascades
Blue Ridge
Chihuahuan Deserts
Sierra Nevada
Texas Blackland Prairies
Madrean Archipelago
Edwards Plateau
Southwestern Appalachians
Ouachita Mountains
Mississippi Valley Loess Plains
East Central Texas Plains
Mojave Basin and Range
Piedmont
Sonoran Basin and Range
South Central Plains
Mississippi Alluvial Plain
Southern Texas Plains
Willamette Valley
Western Gulf Coastal Plain
Coast Range
Klamath Mountains/California High North Coast Range
Central California Valley
Middle Atlantic Coastal Plain
Southern California Mountains
Southeastern Plains
Southern California/Northern Baja Coast
Central California Foothills and Coastal Mountains
Southern Coastal Plain
Southern Florida Coastal Plain
0
2
4
6
Change in MCMT

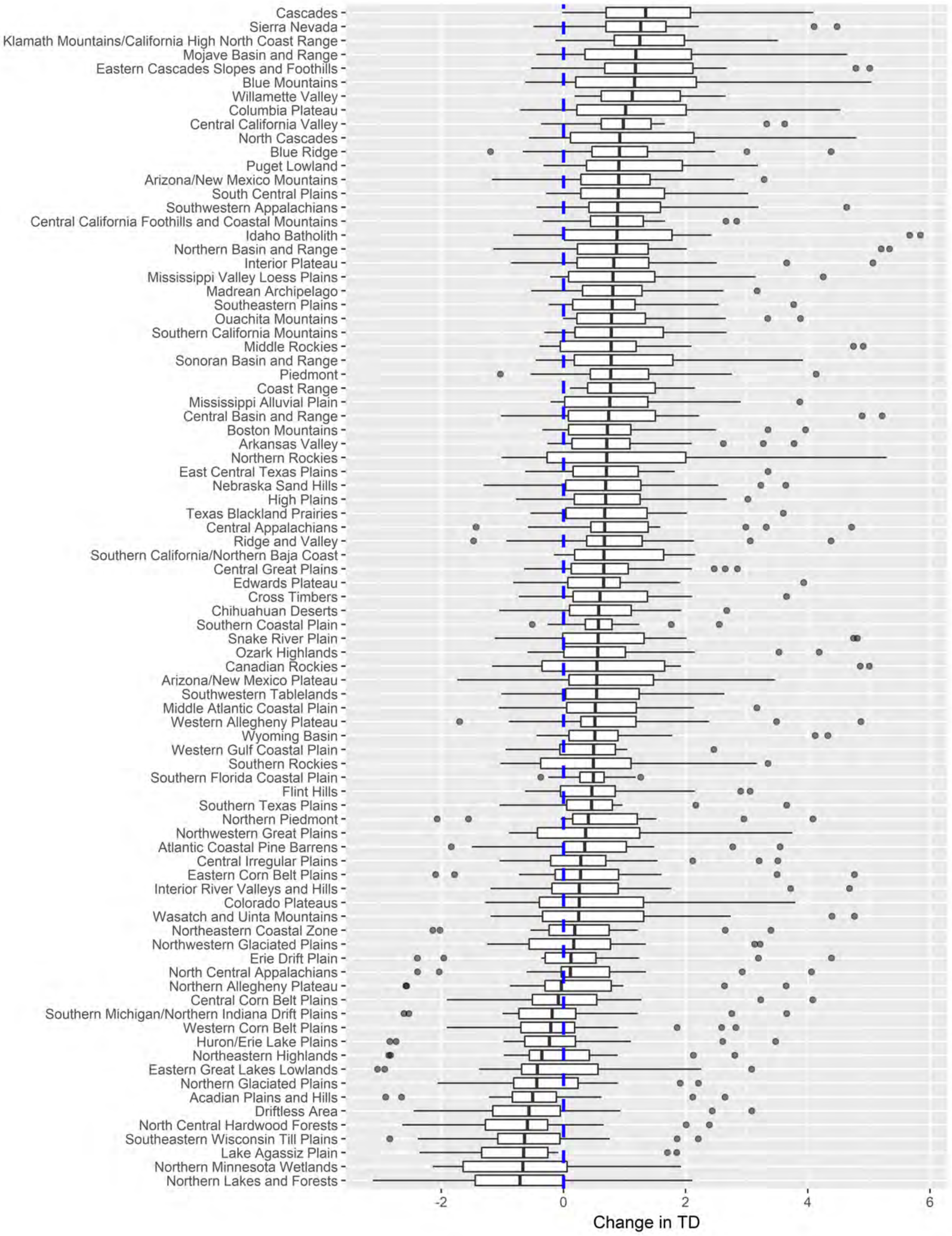
Cascades
Sierra Nevada
Klamath Mountains/California High North Coast Range
Mojave Basin and Range
Eastern Cascades Slopes and Foothills
Blue Mountains
Willamette Valley
Columbia Plateau
Central California Valley
North Cascades
Blue Ridge
Puget Lowland
Arizona/New Mexico Mountains
South Central Plains
Southwestern Appalachians
Central California Foothills and Coastal Mountains
Idaho Batholith
Northern Basin and Range
Interior Plateau
Mississippi Valley Loess Plains
Madrean Archipelago
Southeastern Plains
Ouachita Mountains
Southern California Mountains
Middle Rockies
Sonoran Basin and Range
Piedmont
Coast Range
Mississippi Alluvial Plain
Central Basin and Range
Boston Mountains
Arkansas Valley
Northern Rockies
East Central Texas Plains
Nebraska Sand Hills
High Plains
Texas Blackland Prairies
Central Appalachians
Ridge and Valley
Southern California/Northern Baja Coast
Central Great Plains
Edwards Plateau
Cross Timbers
Chihuahuan Deserts
Southern Coastal Plain
Snake River Plain
Ozark Highlands
Canadian Rockies
Arizona/New Mexico Plateau
Southwestern Tablelands
Middle Atlantic Coastal Plain
Western Allegheny Plateau
Wyoming Basin
Western Gulf Coastal Plain
Southern Rockies
Southern Florida Coastal Plain
Flint Hills
Southern Texas Plains
Northern Piedmont
Northwestern Great Plains
Atlantic Coastal Pine Barrens
Central Irregular Plains
Eastern Corn Belt Plains
Interior River Valleys and Hills
Colorado Plateaus
Wasatch and Uinta Mountains
Northeastern Coastal Zone
Northwestern Glaciated Plains
Erie Drift Plain
North Central Appalachians
Northern Allegheny Plateau
Central Corn Belt Plains
Southern Michigan/Northern Indiana Drift Plains
Western Corn Belt Plains
Huron/Erie Lake Plains
Northeastern Highlands
Eastern Great Lakes Lowlands
Northern Glaciated Plains
Acadian Plains and Hills
Driftless Area
North Central Hardwood Forests
Southeastern Wisconsin Till Plains
Lake Agassiz Plain
Northern Minnesota Wetlands
Northern Lakes and Forests
-2
0
2
4
6
Change in TD

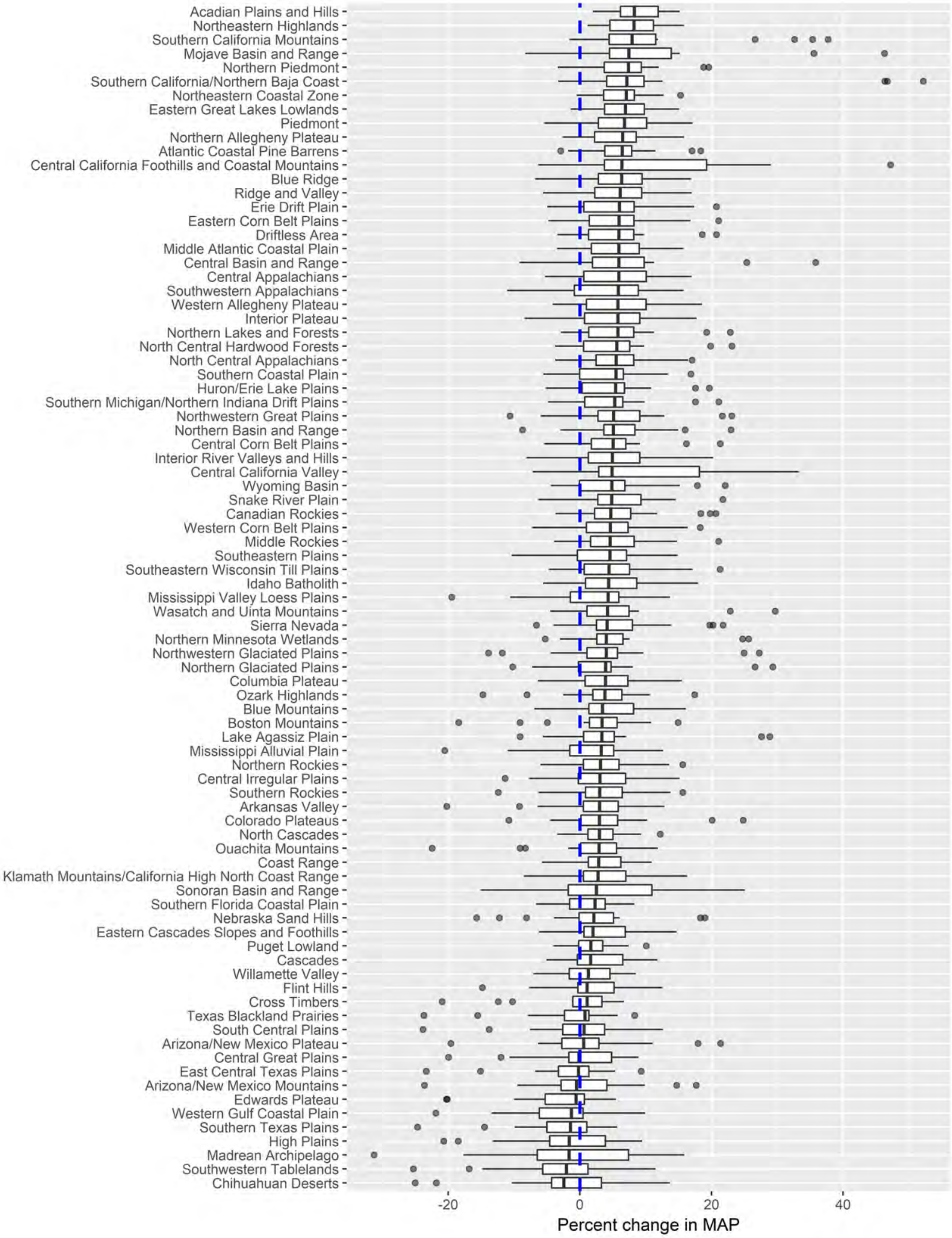
Acadian Plains and Hills
Northeastern Highlands
Southern California Mountains
Mojave Basin and Range
Northern Piedmont
Southern California/Northern Baja Coast
Northeastern Coastal Zone
Eastern Great Lakes Lowlands
Piedmont
Northern Allegheny Plateau
Atlantic Coastal Pine Barrens
Central California Foothills and Coastal Mountains
Blue Ridge
Ridge and Valley
Erie Drift Plain
Eastern Corn Belt Plains
Driftless Area
Middle Atlantic Coastal Plain
Central Basin and Range
Central Appalachians
Southwestern Appalachians
Western Allegheny Plateau
Interior Plateau
Northern Lakes and Forests
North Central Hardwood Forests
North Central Appalachians
Southern Coastal Plain
Huron/Erie Lake Plains
Southern Michigan/Northern Indiana Drift Plains
Northwestern Great Plains
Northern Basin and Range
Central Corn Belt Plains
Interior River Valleys and Hills
Central California Valley
Wyoming Basin
Snake River Plain
Canadian Rockies
Western Corn Belt Plains
Middle Rockies
Southeastern Plains
Southeastern Wisconsin Till Plains
Idaho Batholith
Mississippi Valley Loess Plains
Wasatch and Uinta Mountains
Sierra Nevada
Northern Minnesota Wetlands
Northwestern Glaciated Plains
Northern Glaciated Plains
Columbia Plateau
Ozark Highlands
Blue Mountains
Boston Mountains
Lake Agassiz Plain
Mississippi Alluvial Plain
Northern Rockies
Central Irregular Plains
Southern Rockies
Arkansas Valley
Colorado Plateaus
North Cascades
Ouachita Mountains
Coast Range
Klamath Mountains/California High North Coast Range
Sonoran Basin and Range
Southern Florida Coastal Plain
Nebraska Sand Hills
Eastern Cascades Slopes and Foothills
Puget Lowland
Cascades
Willamette Valley
Flint Hills
Cross Timbers
Texas Blackland Prairies
South Central Plains
Arizona/New Mexico Plateau
Central Great Plains
East Central Texas Plains
Arizona/New Mexico Mountains
Edwards Plateau
Western Gulf Coastal Plain
Southern Texas Plains
High Plains
Madrean Archipelago
Southwestern Tablelands
Chihuahuan Deserts
-20
0
20
40
Percent change in MAP

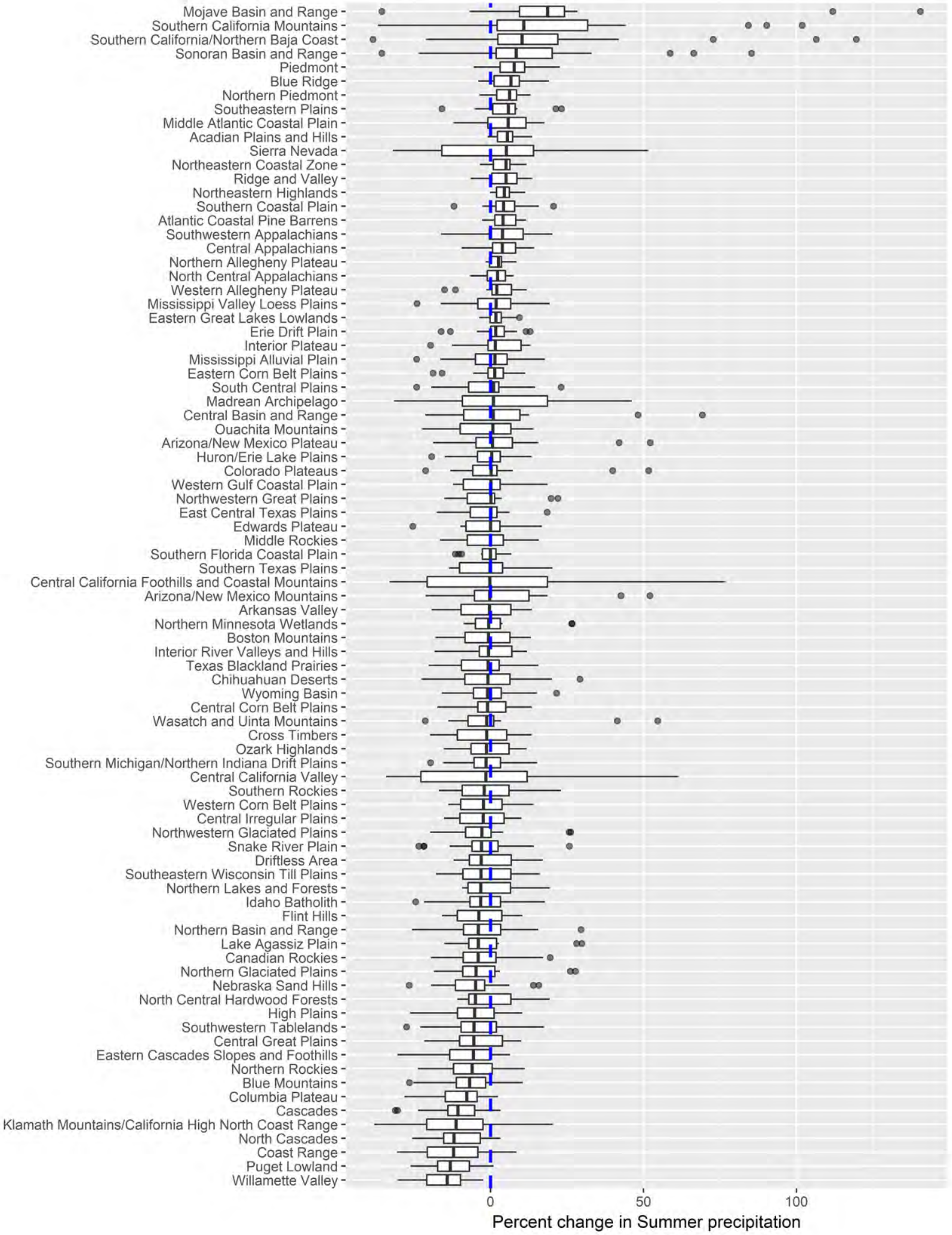
Mojave Basin and Range
Southern California Mountains
Southern California/Northern Baja Coast
Sonoran Basin and Range
Piedmont
Blue Ridge
Northern Piedmont
Southeastern Plains
Middle Atlantic Coastal Plain
Acadian Plains and Hills
Sierra Nevada
Northeastern Coastal Zone
Ridge and Valley
Northeastern Highlands
Southern Coastal Plain
Atlantic Coastal Pine Barrens
Southwestern Appalachians
Central Appalachians
Northern Allegheny Plateau
North Central Appalachians
Western Allegheny Plateau
Mississippi Valley Loess Plains
Eastern Great Lakes Lowlands
Erie Drift Plain
Interior Plateau
Mississippi Alluvial Plain
Eastern Corn Belt Plains
South Central Plains
Madrean Archipelago
Central Basin and Range
Ouachita Mountains
Arizona/New Mexico Plateau
Huron/Erie Lake Plains
Colorado Plateaus
Western Gulf Coastal Plain
Northwestern Great Plains
East Central Texas Plains
Edwards Plateau
Middle Rockies
Southern Florida Coastal Plain
Southern Texas Plains
Central California Foothills and Coastal Mountains
Arizona/New Mexico Mountains
Arkansas Valley
Northern Minnesota Wetlands
Boston Mountains
Interior River Valleys and Hills
Texas Blackland Prairies
Chihuahuan Deserts
Wyoming Basin
Central Corn Belt Plains
Wasatch and Uinta Mountains
Cross Timbers
Ozark Highlands
Southern Michigan/Northern Indiana Drift Plains
Central California Valley
Southern Rockies
Western Corn Belt Plains
Central Irregular Plains
Northwestern Glaciated Plains
Snake River Plain
Driftless Area
Southeastern Wisconsin Till Plains
Northern Lakes and Forests
Idaho Batholith
Flint Hills
Northern Basin and Range
Lake Agassiz Plain
Canadian Rockies
Northern Glaciated Plains
Nebraska Sand Hills
North Central Hardwood Forests
High Plains
Southwestern Tablelands
Central Great Plains
Eastern Cascades Slopes and Foothills
Northern Rockies
Blue Mountains
Columbia Plateau
Cascades
Klamath Mountains/California High North Coast Range
North Cascades
Coast Range
Puget Lowland
Willamette Valley
0
50
100
Percent change in Summer precipitation

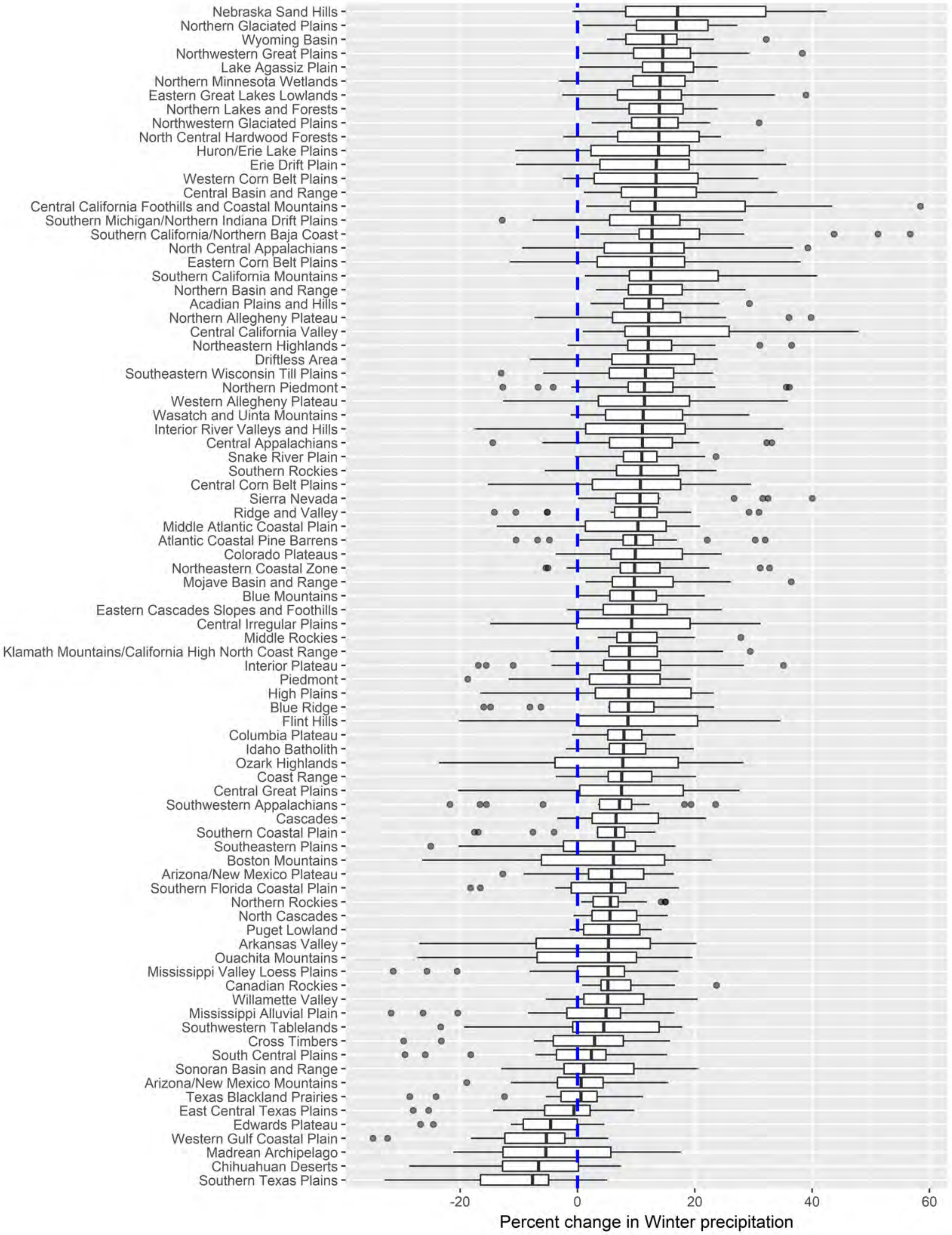
Nebraska Sand Hills
Northern Glaciated Plains
Wyoming Basin
Northwestern Great Plains
Lake Agassiz Plain
Northern Minnesota Wetlands
Eastern Great Lakes Lowlands
Northern Lakes and Forests
Northwestern Glaciated Plains
North Central Hardwood Forests
Huron/Erie Lake Plains
Erie Drift Plain
Western Corn Belt Plains
Central Basin and Range
Central California Foothills and Coastal Mountains
Southern Michigan/Northern Indiana Drift Plains
Southern California/Northern Baja Coast
North Central Appalachians
Eastern Corn Belt Plains
Southern California Mountains
Northern Basin and Range
Acadian Plains and Hills
Northern Allegheny Plateau
Central California Valley
Northeastern Highlands
Driftless Area
Southeastern Wisconsin Till Plains
Northern Piedmont
Western Allegheny Plateau
Wasatch and Uinta Mountains
Interior River Valleys and Hills
Central Appalachians
Snake River Plain
Southern Rockies
Central Corn Belt Plains
Sierra Nevada
Ridge and Valley
Middle Atlantic Coastal Plain
Atlantic Coastal Pine Barrens
Colorado Plateaus
Northeastern Coastal Zone
Mojave Basin and Range
Blue Mountains
Eastern Cascades Slopes and Foothills
Central Irregular Plains
Middle Rockies
Klamath Mountains/California High North Coast Range
Interior Plateau
Piedmont
High Plains
Blue Ridge
Flint Hills
Columbia Plateau
Idaho Batholith
Ozark Highlands
Coast Range
Central Great Plains
Southwestern Appalachians
Cascades
Southern Coastal Plain
Southeastern Plains
Boston Mountains
Arizona/New Mexico Plateau
Southern Florida Coastal Plain
Northern Rockies
North Cascades
Puget Lowland
Arkansas Valley
Ouachita Mountains
Mississippi Valley Loess Plains
Canadian Rockies
Willamette Valley
Mississippi Alluvial Plain
Southwestern Tablelands
Cross Timbers
South Central Plains
Sonoran Basin and Range
Arizona/New Mexico Mountains
Texas Blackland Prairies
East Central Texas Plains
Edwards Plateau
Western Gulf Coastal Plain
Madrean Archipelago
Chihuahuan Deserts
Southern Texas Plains
-20
0
20
40
60
Percent change in Winter precipitation

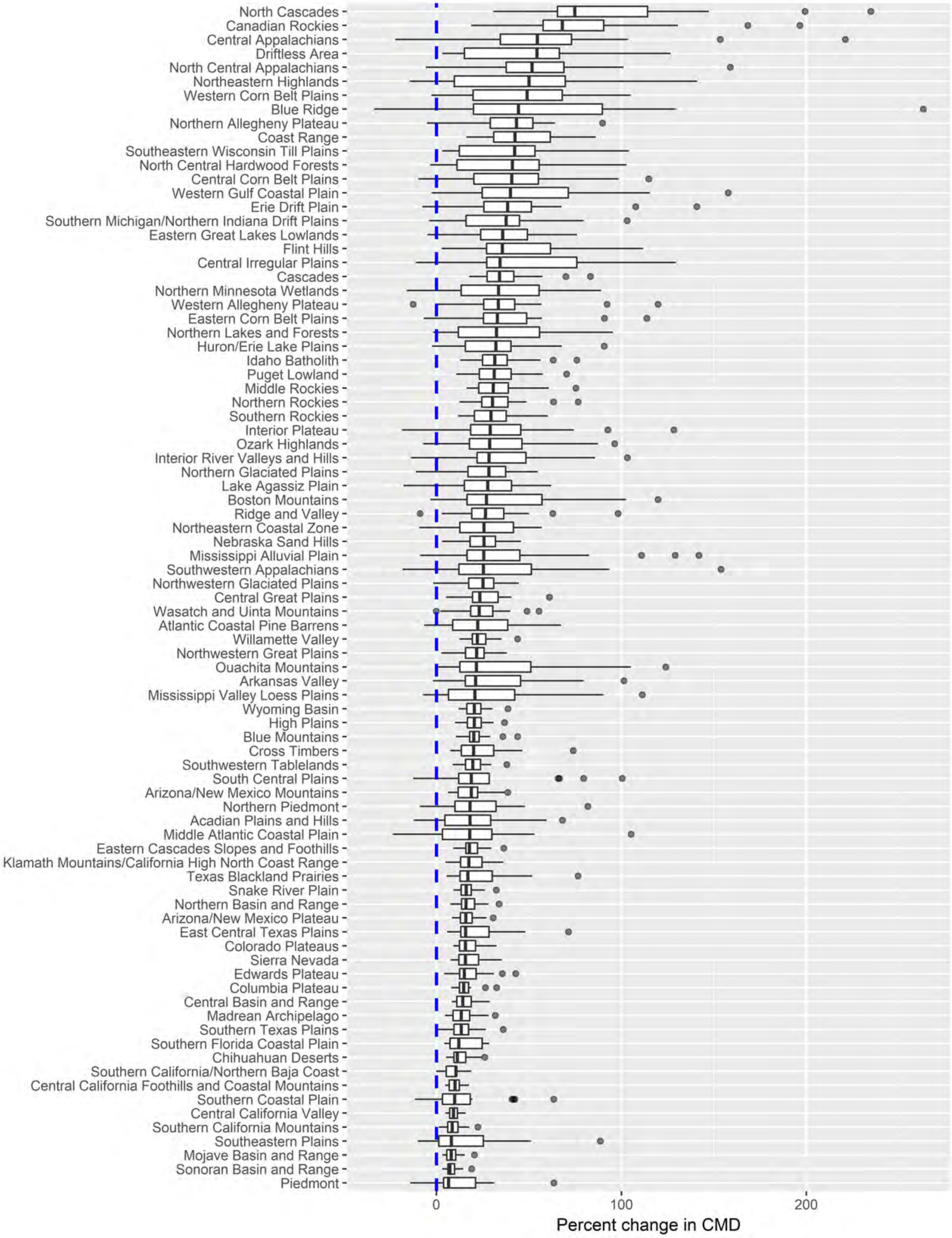
North Cascades
Canadian Rockies
Central Appalachians
Driftless Area
North Central Appalachians
Northeastern Highlands
Western Corn Belt Plains
Blue Ridge
Northern Allegheny Plateau
Coast Range
Southeastern Wisconsin Till Plains
North Central Hardwood Forests
Central Corn Belt Plains
Western Gulf Coastal Plain
Erie Drift Plain
Southern Michigan/Northern Indiana Drift Plains
Eastern Great Lakes Lowlands
Flint Hills
Central Irregular Plains
Cascades
Northern Minnesota Wetlands
Western Allegheny Plateau
Eastern Corn Belt Plains
Northern Lakes and Forests
Huron/Erie Lake Plains
Idaho Batholith
Puget Lowland
Middle Rockies
Northern Rockies
Southern Rockies
Interior Plateau
Ozark Highlands
Interior River Valleys and Hills
Northern Glaciated Plains
Lake Agassiz Plain
Boston Mountains
Ridge and Valley
Northeastern Coastal Zone
Nebraska Sand Hills
Mississippi Alluvial Plain
Southwestern Appalachians
Northwestern Glaciated Plains
Central Great Plains
Wasatch and Uinta Mountains
Atlantic Coastal Pine Barrens
Willamette Valley
Northwestern Great Plains
Ouachita Mountains
Arkansas Valley
Mississippi Valley Loess Plains
Wyoming Basin
High Plains
Blue Mountains
Cross Timbers
Southwestern Tablelands
South Central Plains
Arizona/New Mexico Mountains
Northern Piedmont
Acadian Plains and Hills
Middle Atlantic Coastal Plain
Eastern Cascades Slopes and Foothills
Klamath Mountains/California High North Coast Range
Texas Blackland Prairies
Snake River Plain
Northern Basin and Range
Arizona/New Mexico Plateau
East Central Texas Plains
Colorado Plateaus
Sierra Nevada
Edwards Plateau
Columbia Plateau
Central Basin and Range
Madrean Archipelago
Southern Texas Plains
Southern Florida Coastal Plain
Chihuahuan Deserts
Southern California/Northern Baja Coast
Central California Foothills and Coastal Mountains
Southern Coastal Plain
Central California Valley
Southern California Mountains
Southeastern Plains
Mojave Basin and Range
Sonoran Basin and Range
Piedmont
0
100
200
Percent change in CMD

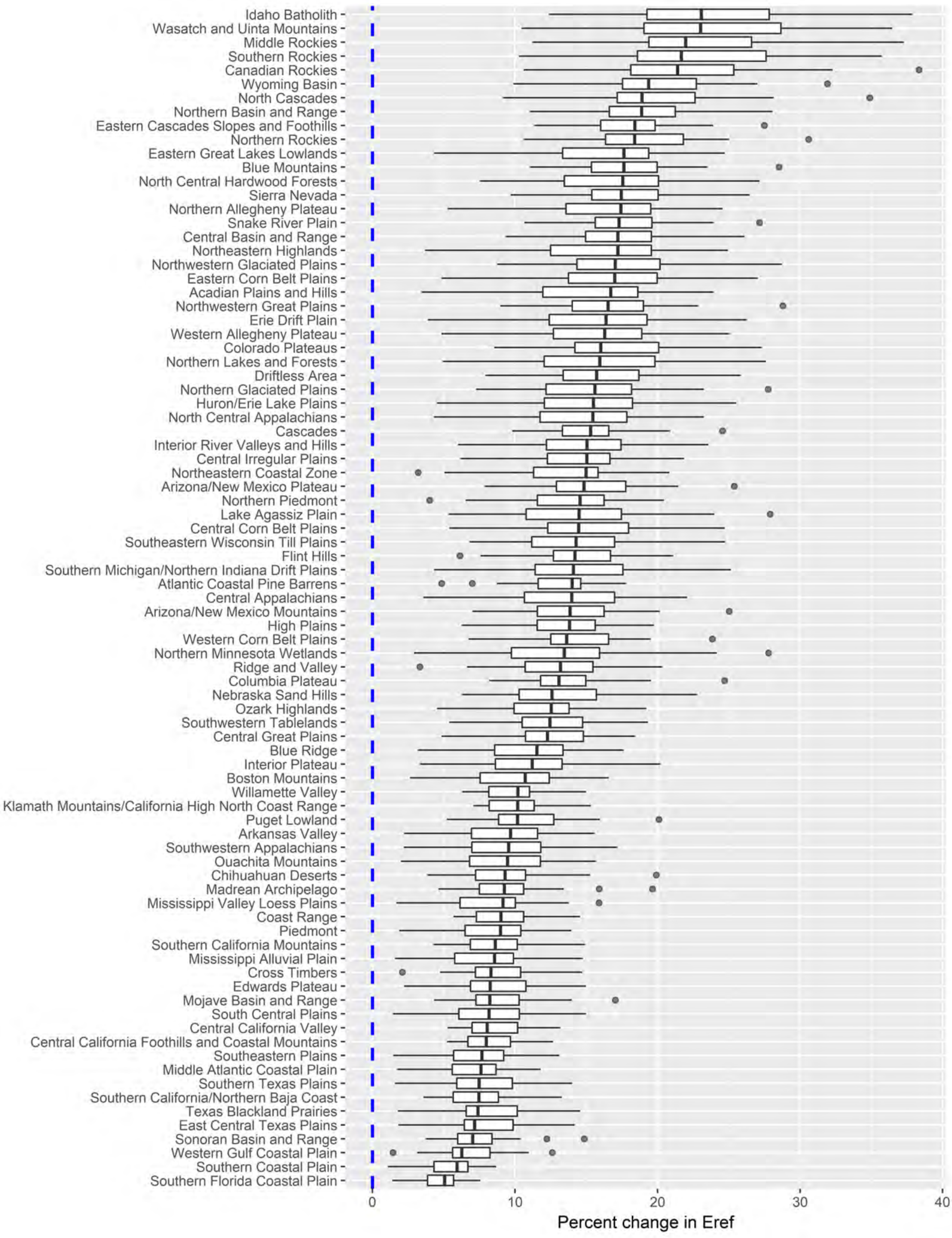
Idaho Batholith
Wasatch and Uinta Mountains
Middle Rockies
Southern Rockies
Canadian Rockies
Wyoming Basin
North Cascades
Northern Basin and Range
Eastern Cascades Slopes and Foothills
Northern Rockies
Eastern Great Lakes Lowlands
Blue Mountains
North Central Hardwood Forests
Sierra Nevada
Northern Allegheny Plateau
Snake River Plain
Central Basin and Range
Northeastern Highlands
Northwestern Glaciated Plains
Eastern Corn Belt Plains
Acadian Plains and Hills
Northwestern Great Plains
Erie Drift Plain
Western Allegheny Plateau
Colorado Plateaus
Northern Lakes and Forests
Driftless Area
Northern Glaciated Plains
Huron/Erie Lake Plains
North Central Appalachians
Cascades
Interior River Valleys and Hills
Central Irregular Plains
Northeastern Coastal Zone
Arizona/New Mexico Plateau
Northern Piedmont
Lake Agassiz Plain
Central Corn Belt Plains
Southeastern Wisconsin Till Plains
Flint Hills
Southern Michigan/Northern Indiana Drift Plains
Atlantic Coastal Pine Barrens
Central Appalachians
Arizona/New Mexico Mountains
High Plains
Western Corn Belt Plains
Northern Minnesota Wetlands
Ridge and Valley
Columbia Plateau
Nebraska Sand Hills
Ozark Highlands
Southwestern Tablelands
Central Great Plains
Blue Ridge
Interior Plateau
Boston Mountains
Willamette Valley
Klamath Mountains/California High North Coast Range
Puget Lowland
Arkansas Valley
Southwestern Appalachians
Ouachita Mountains
Chihuahuan Deserts
Madrean Archipelago
Mississippi Valley Loess Plains
Coast Range
Piedmont
Southern California Mountains
Mississippi Alluvial Plain
Cross Timbers
Edwards Plateau
Mojave Basin and Range
South Central Plains
Central California Valley
Central California Foothills and Coastal Mountains
Southeastern Plains
Middle Atlantic Coastal Plain
Southern Texas Plains
Southern California/Northern Baja Coast
Texas Blackland Prairies
East Central Texas Plains
Sonoran Basin and Range
Western Gulf Coastal Plain
Southern Coastal Plain
Southern Florida Coastal Plain
0
10
20
30
40
Percent change in Eref

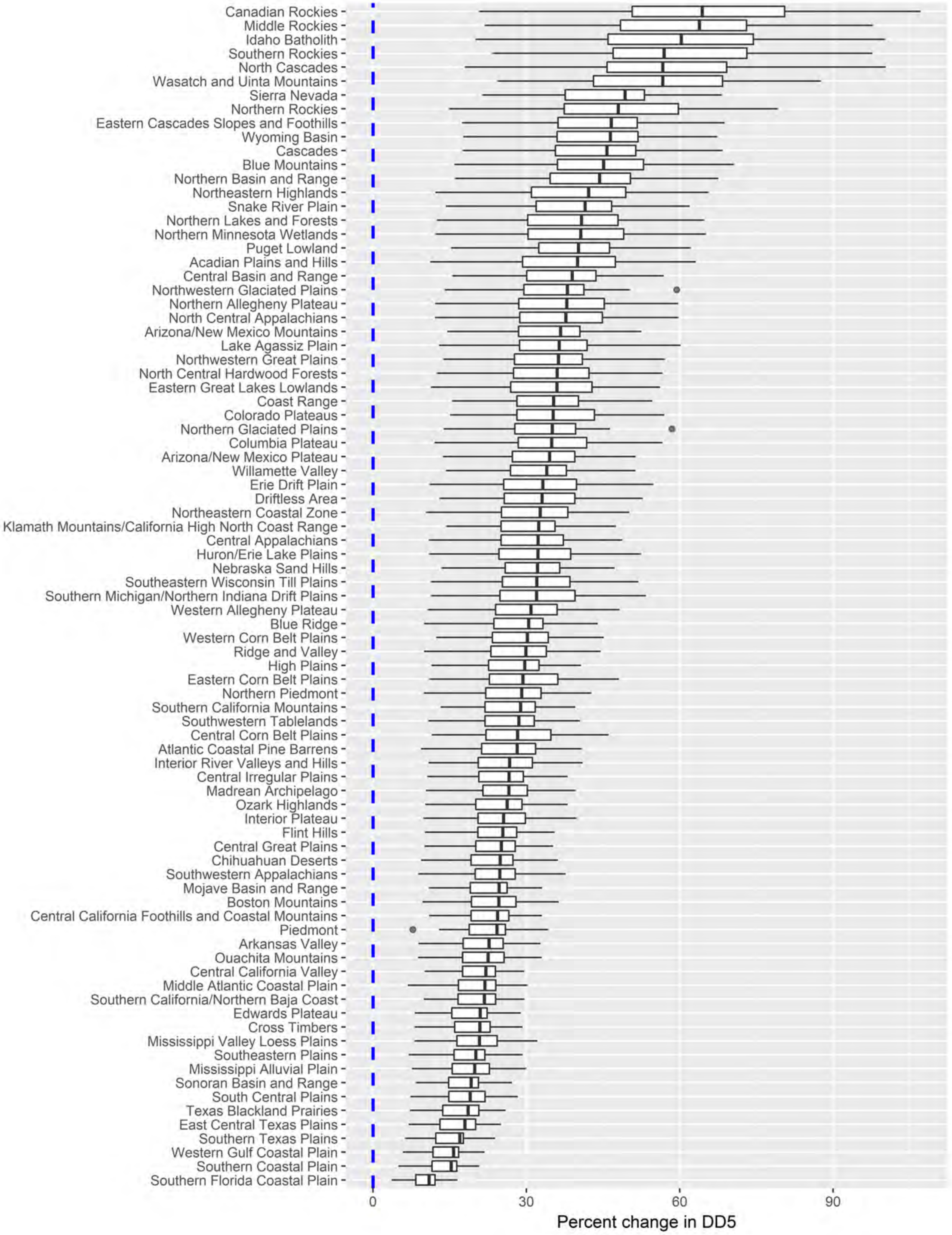
Canadian Rockies
Middle Rockies
Idaho Batholith
Southern Rockies
North Cascades
Wasatch and Uinta Mountains
Sierra Nevada
Northern Rockies
Eastern Cascades Slopes and Foothills
Wyoming Basin
Cascades
Blue Mountains
Northern Basin and Range
Northeastern Highlands
Snake River Plain
Northern Lakes and Forests
Northern Minnesota Wetlands
Puget Lowland
Acadian Plains and Hills
Central Basin and Range
Northwestern Glaciated Plains
Northern Allegheny Plateau
North Central Appalachians
Arizona/New Mexico Mountains
Lake Agassiz Plain
Northwestern Great Plains
North Central Hardwood Forests
Eastern Great Lakes Lowlands
Coast Range
Colorado Plateaus
Northern Glaciated Plains
Columbia Plateau
Arizona/New Mexico Plateau
Willamette Valley
Erie Drift Plain
Driftless Area
Northeastern Coastal Zone
Klamath Mountains/California High North Coast Range
Central Appalachians
Huron/Erie Lake Plains
Nebraska Sand Hills
Southeastern Wisconsin Till Plains
Southern Michigan/Northern Indiana Drift Plains
Western Allegheny Plateau
Blue Ridge
Western Corn Belt Plains
Ridge and Valley
High Plains
Eastern Corn Belt Plains
Northern Piedmont
Southern California Mountains
Southwestern Tablelands
Central Corn Belt Plains
Atlantic Coastal Pine Barrens
Interior River Valleys and Hills
Central Irregular Plains
Madrean Archipelago
Ozark Highlands
Interior Plateau
Flint Hills
Central Great Plains
Chihuahuan Deserts
Southwestern Appalachians
Mojave Basin and Range
Boston Mountains
Central California Foothills and Coastal Mountains
Piedmont
Arkansas Valley
Ouachita Mountains
Central California Valley
Middle Atlantic Coastal Plain
Southern California/Northern Baja Coast
Edwards Plateau
Cross Timbers
Mississippi Valley Loess Plains
Southeastern Plains
Mississippi Alluvial Plain
Sonoran Basin and Range
South Central Plains
Texas Blackland Prairies
East Central Texas Plains
Southern Texas Plains
Western Gulf Coastal Plain
Southern Coastal Plain
Southern Florida Coastal Plain
0
30
60
90
Percent change in DD5

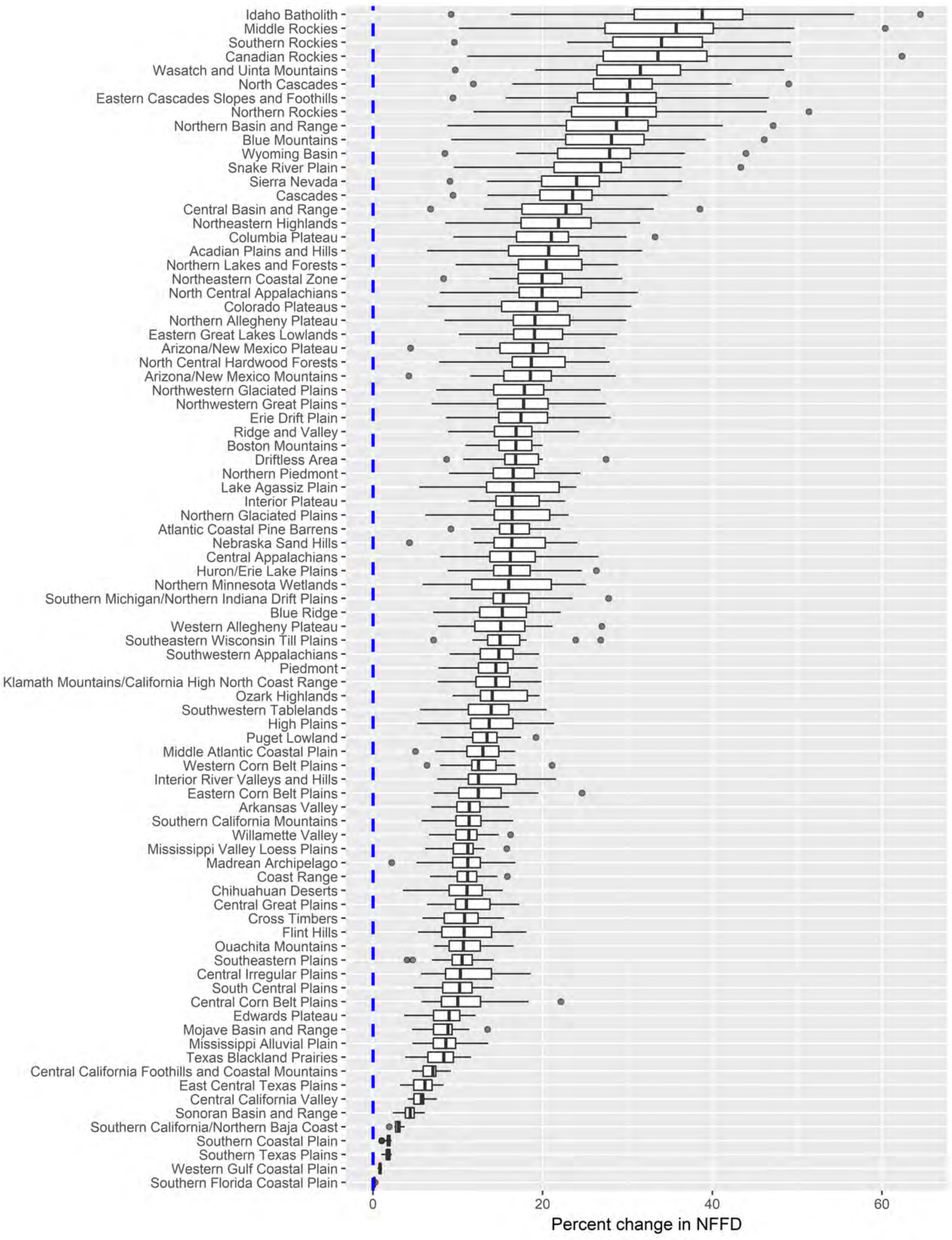

Idaho Batholith
Middle Rockies
Southern Rockies
Canadian Rockies
Wasatch and Uinta Mountains
North Cascades
Eastern Cascades Slopes and Foothills
Northern Rockies
Northern Basin and Range
Blue Mountains
Wyoming Basin
Snake River Plain
Sierra Nevada
Cascades
Central Basin and Range
Northeastern Highlands
Columbia Plateau
Acadian Plains and Hills
Northern Lakes and Forests
Northeastern Coastal Zone
North Central Appalachians
Colorado Plateaus
Northern Allegheny Plateau
Eastern Great Lakes Lowlands
Arizona/New Mexico Plateau
North Central Hardwood Forests
Arizona/New Mexico Mountains
Northwestern Glaciated Plains
Northwestern Great Plains
Erie Drift Plain
Ridge and Valley
Boston Mountains
Driftless Area
Northern Piedmont
Lake Agassiz Plain
Interior Plateau
Northern Glaciated Plains
Atlantic Coastal Pine Barrens
Nebraska Sand Hills
Central Appalachians
Huron/Erie Lake Plains
Northern Minnesota Wetlands
Southern Michigan/Northern Indiana Drift Plains
Blue Ridge
Western Allegheny Plateau
Southeastern Wisconsin Till Plains
Southwestern Appalachians
Piedmont
Klamath Mountains/California High North Coast Range
Ozark Highlands
Southwestern Tablelands
High Plains
Puget Lowland
Middle Atlantic Coastal Plain
Western Corn Belt Plains
Interior River Valleys and Hills
Eastern Corn Belt Plains
Arkansas Valley
Southern California Mountains
Willamette Valley
Mississippi Valley Loess Plains
Madrean Archipelago
Coast Range
Chihuahuan Deserts
Central Great Plains
Cross Timbers
Flint Hills
Ouachita Mountains
Southeastern Plains
Central Irregular Plains
South Central Plains
Central Corn Belt Plains
Edwards Plateau
Mojave Basin and Range
Mississippi Alluvial Plain
Texas Blackland Prairies
Central California Foothills and Coastal Mountains
East Central Texas Plains
Central California Valley
Sonoran Basin and Range
Southern California/Northern Baja Coast
Southern Coastal Plain
Southern Texas Plains
Western Gulf Coastal Plain
Southern Florida Coastal Plain
0
20
40
60
Percent change in NFFD

References

1. Moilanen, A., Wintley, B. A., Elith, J. & Burgman, M. Uncertainty analysis for regional-scale reserve selection. *Conservation Biology* **20,** 1688–1697 (2006).

2. Kujala, H., Moilanen, A., Araújo, M. B. & Cabeza, M. Conservation planning with uncertain climate change projections. *PLoS ONE* **8,** (2013).

3. Carroll, C. *et al.* Scale-dependent complementarity of climatic velocity and environmental diversity for identifying priority areas for conservation under climate change. *Global Change Biology* (2017). doi:10.1111/gcb.13679

4. Carroll, C., Dunk, J. R. & Moilanen, A. Optimizing resiliency of reserve networks to climate change: multispecies conservation planning in the Pacific Northwest, USA. *Global Change Biology* **16,** 891–904 (2010).

5. Omernik, J. M. & Griffith, G. E. Ecoregions of the conterminous United States: evolution of a hierarchical spatial framework. *Environmental management* **54,** 1249–66 (2014).
